# Using a co-created checklist to improve on-farm biosecurity: an observational pilot intervention with pig farmers and livestock field officers in Sumbawanga, Tanzania

**DOI:** 10.3389/fvets.2025.1567072

**Published:** 2025-07-15

**Authors:** Aashima Auplish, Kuboja Mjuberi, Henry Magwisha, Damian Tago, Anica Buckel, Ugo Pica Ciamarra, Melissa Mclaws, Martin Heilmann

**Affiliations:** ^1^The Food and Agriculture Organization of the United Nations (FAO) Headquarters, Rome, Italy; ^2^The Food and Agriculture Organization of the United Nations (FAO) Representation in United Republic of Tanzania, Dar es Salaam, Tanzania; ^3^The Ministry of Livestock and Fisheries, United Republic of Tanzania, Sumbawanga, Tanzania

**Keywords:** biosecurity, pig production, Tanzania, participatory approach, co-creation, Theoretical Domains Framework, evaluation

## Abstract

The Tanzanian pig sector has the capacity to become market-oriented but it is constrained by significant factors like poor husbandry, management practices and disease, like African swine fever (ASF). Good biosecurity is essential to prevent, minimise or even eliminate biosecurity risks on farms. This study aimed to evaluate a pilot intervention based on an innovative, participatory approach to progressively improve biosecurity practices on small- and medium-scale pig farms in Tanzania. An observational study was conducted, where 30 farms were systematically monitored to assess the impact of using a co-created checklist on biosecurity compliance and production parameters. Livestock field officers (LFOs) were trained to provide technical guidance to farmers to implement the checklist. Focus group discussions (FGDs) were also conducted with LFOs, which were coded and thematically analysed. The median compliance score for biosecurity was significantly higher after the pilot intervention (20.0 out of 26 practices or 76.9%) compared to baseline (median of 5.50 out of 26 practices 21.2%). The time spent implementing biosecurity per sow (per day) increased from a median of 7.8–18.6 min by the end of the intervention. Pre-weaning mortality decreased from 28.6 to 25.0% and cost of antimicrobial use per sow (per month) was reduced by 57%. Meanwhile, FGDs revealed that the pilot intervention allowed LFOs to connect with farmers to provide services and collaborate with other LFOs to co-develop solutions for farmers. Despite an initial lack of trust, the relationships between LFOs and farmers were described to have positively transformed. These findings highlight the potential of using bottom-up approaches, combined with sensitisation and capacity-building, to address the unique challenges of biosecurity in low-resource settings.

## 1 Introduction

In Tanzania, about 537,000 (3.7%) households raise pigs and there are approximately 3.2 million pigs distributed throughout the country (1). Although the Tanzanian pig sector has huge potential to grow, the sector is challenged by inadequate extension services, poor husbandry and slaughtering practices, limited marketing infrastructure and diseases such as African swine fever (ASF), which represents one of the biggest constraints to pig farming in the country (2).

Many outbreaks of transboundary animal diseases (TAD) and/or production limiting diseases are spread by human actions and indirect transmission, such as the movement of infected animals to the farm, sharing infected breeding animals, using contaminated feed, and poor disposal of infected waste that may be spread by animals like birds and dogs (3). Good biosecurity is essential to prevent, minimise or even eliminate biosecurity risks on farms. For instance, it has been predicted that biosecurity implemented within 14 days of the onset of an outbreak can avert up to 74% of ASF-related deaths in pigs (4).

To curb production-limiting disease outbreaks and more broadly, improve pig farming in Tanzania, the Government of Tanzania and the Food and Agriculture Organization of the United Nations (FAO) have collaborated to implement the ‘Progressive Management Pathway for Terrestrial Animal Biosecurity’ (PMP-TAB), which is a stepwise approach to improve biosecurity along value chains and ultimately strengthen livestock systems, sustainably (5). The FAO defines the term ‘biosecurity’ as a strategic and integrated approach to analysing and managing risks to human, animal and plant life and health, and associated risks to the environment. It is a holistic concept that encompasses health policy, regulation and practices to protect agriculture, food and the environment from biological risks (6). The stepwise approach starts with identifying the practices, risks, interests and benefits; improving biosecurity at farm level and after successful piloting, the approach will be scaled up to other value chain nodes and/or livestock systems or to other geographic areas (5). This approach responds to the existing challenge of the poor uptake of recommended biosecurity practices by value chain actors, especially in low resource settings, such as rural areas in low and middle income countries (7).

In fact, improving biosecurity and its uptake is often challenging by due to the “knowledge-action gap.” Consistent with broader findings on the intention-behaviour gap in the social and behavioural sciences, simply possessing knowledge of benefits or forming the intention to act are frequently poor predictors of whether farmers consistently implement biosecurity measures (8–15). Consequently, achieving sustainable improvements in on-farm biosecurity necessitates moving beyond solely information-based strategies towards approaches that emphasise meaningful stakeholder engagement and facilitate behavioural change. This has led to increased interest amongst researchers and practitioners to understand the enablers and barriers related to disease control and prevention measures using socio-psychological frameworks (16). These frameworks provide theory driven approaches for identifying and analysing factors influencing behaviours and to design intervention mechanism likely to drive behaviour change. Recognising the importance of systematically understanding these factors for biosecurity uptake in the Tanzanian context, this study incorporated established behavioural science frameworks to explore influencing factors.

This present study takes an innovative approach by utilising the Theoretical Domains Framework (TDF) (17) and the Capability, Opportunity, Motivation-Behaviour (COM-B) Model (18) as well as blended learning concepts with local-level public-private partnerships, participatory approaches, and financial incentives. At the centre at the field level is a partnership between public (government employed) and private livestock officers (LFOs) and farmers who together are responsible for progressively implementing biosecurity practices outlined in an agreed-upon checklist for pig farms. LFOs in Tanzania are trained (at certificate, diploma or bachelor level) in livestock health and production and are responsible for providing extension services at the ward-level.

The aims of this study were to (i) describe the local biosecurity situation of small to medium-scale pig farms in Sumbawanga Municipal Council (MC), Tanzania; (ii) identify perceived challenges, successes and opportunities related to LFOs engaging with farmers to implement the checklist in farms, (iii) identify factors influencing (enabling and disabling factors) farmers’ decision-making around uptake of good biosecurity practices and (iv) investigate if a co-created checklist on biosecurity can lead to progressive improvements of biosecurity and production on pig farms.

## 2 Methods

### 2.1 Study design

An observational baseline-and-endline on-farm pilot intervention was performed from May to October 2024.

### 2.2 Study area

**Figure 1:**
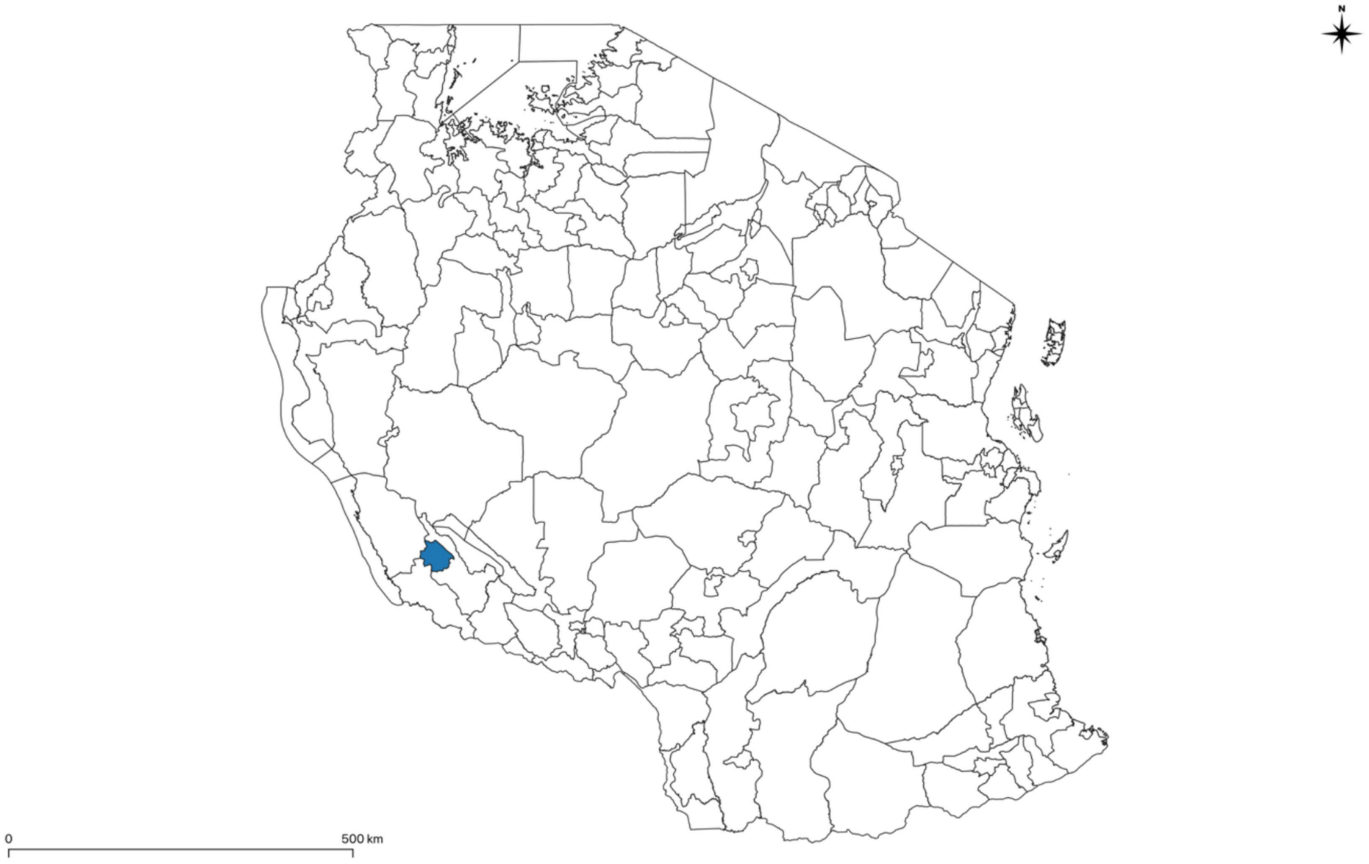
Map of the United Republic of Tanzania (to the district level) with the study location, Sumbawanga MC, indicated by the coloured area. Developed using GADM data. Created and reproduced with permission from GADM. Published under CC BY 4.0 licence.

This study was conducted in Sumbawanga MC, the United Republic of Tanzania (hereinafter referred to as Tanzania), located in East Africa (Figure 1). Tanzania has 30 administrative regions comprising 184 districts, which are subdivided into divisions. Each division is made up of three to five wards, and the lowest administrative units are the villages. Sumbawanga MC is a district in the Rukwa region and has 19 wards. According to the latest available numbers from a study conducted in Sumbawanga MC in 2017, the population of pigs is 13,010 heads and the majority of the farmers are smallholders with less than 10 pigs per herd (19).

### 2.3 Study population

**Figure 2:**
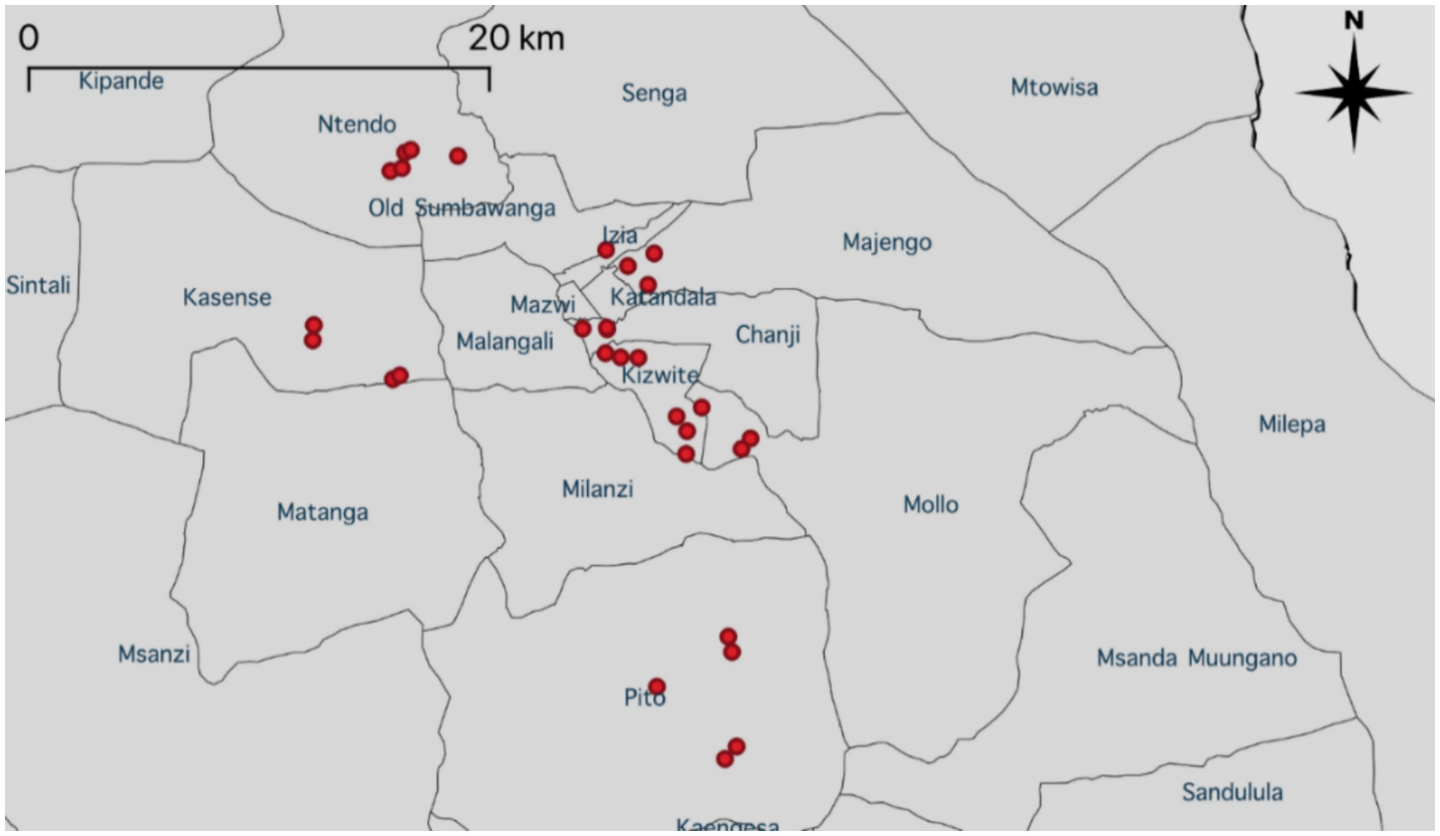
Partial map of the United Republic of Tanzania indicating locations of selected farms in red (*n* = 30) and their respective wards in Sumbawanga MC.

The study population included pig farmers in all 19 wards of Sumbawanga MC (Figures 1, 2). Reliable information about the number and location of pig farms (i.e., a reliable sampling frame) was not available prior to this study. Instead, pig farms were identified by LFOs based on local knowledge of their respective wards. Geospatial data related to the farm location was exported from Kobo Collect and imported into QGIS (version 3.26.1) to create maps. The maps were developed using data from GADM (20). A total of 30 farms were purposively selected for monthly systematic monitoring of compliance with the checklist on biosecurity (21). In addition to systematic monitoring, LFOs could conduct voluntary audits to other farms on an ad-hoc basis, provided farmers were open to regular visits and implementing biosecurity measures.

### 2.4 Pilot intervention

The pilot intervention was centred around the implementation of a co-created checklist on biosecurity with small- and medium-scale pig farmers who expressed a willingness to participate. The checklist outlining 26 biosecurity practices was previously designed using participatory methods, specifically co-creation (22). Each practice was further detailed with examples of indicators to make sure local stakeholders have a clear understanding of how it is implemented (for instance, the practice related to access control can comprise different elements like a fence, security guard, sign indicating restricted access, etc.). The checklist considers national legislation, includes indicators to measure compliance, and was validated by local stakeholders (specifically, farmers and farm labourers) and subject matter experts to ensure that it is practical and tailored for the local context of Sumbawanga. The methodology used to develop the checklist has been published previously in detail (21). Participatory approaches were utilised given the existing challenges of traditional approaches to implement biosecurity such as a mismatch between central and local levels, neglection of prerequisites, unsuitable standards for small-scale actors and a limited focus on biosecurity in low- and middle-income countries (23). In line with the participatory approach, the pilot intervention activities in Sumbawanga were co-created with relevant stakeholders.

A blended capacity building approach (using in-person and online training methods) was used to train 14 public and five private LFOs to provide extension to pig farmers to implement the biosecurity checklist on their farms (i.e., perform biosecurity audits of farms). All LFOs from Sumbawanga MC were invited to attend the training and participate in the pilot study as enumerators. Training was delivered on a monthly basis from April to July and the training sessions were 3 days in duration. LFOs were then encouraged to voluntarily visit and audit farms. During each audit by an LFO, farmers were encouraged to improve biosecurity by first focusing on a few self-selected practices from the checklist and then, progressively improving adoption of practices. Two sensitisation events were held in Sumbawanga to (i) foster collaboration and knowledge exchange between farmers and LFOs about the checklist and (ii) recognise participating farmers with higher levels of compliance.

### 2.5 Data collection

#### 2.5.1 Quantitative

##### 2.5.1.1 Systematic monitoring

Monthly systematic monitoring of compliance to the biosecurity checklist was undertaken on 30 pig farms between May and October 2024 (Figure 2). The checklist was uploaded as a survey to Kobo Collect (version 2024.1.3) to use for auditing purposes and a local enumerator was trained by the authors (AA and MH) to ensure a standardised approach to data collection. Kobo Collect is part of the Kobo Toolbox, an open-source and user-friendly platform for researchers looking to streamline their data collection process (24). Likewise, LFOs were requested to collect data using Kobo Collect during ad-hoc farm visits. Data collected from farms was based on direct observation by the enumerator(s), discussion with farmers and farm records (if available).

Each of the 30 selected farmers signed a community contract to signify their commitment to progressive adoption of biosecurity practices on farms and to consent to data collection. This approach leverages evidence from previous studies demonstrating the effectiveness of psychological commitment mechanisms in encouraging adoption (25). Obtained data was treated anonymously without using any personal identifiable information. As an approved field project under government oversight and focused on implementation evaluation, no further oversight was indicated.

Data relating to compliance to biosecurity practices, production parameters, antimicrobial use and labour time were collected regularly. During the final phase of monitoring (i.e., endline), farmers were also asked survey questions about community and individual level factors influencing uptake of biosecurity practices informed by the Theoretical Domains Framework (TDF) (17). The TDF provides a systematic way to identify determinants of behaviour by synthesising constructs from multiple behaviour change theories into 14 domains. These domains map onto the broader COM-B model (18).

##### 2.5.1.2 Ad-hoc monitoring

**Figure 3:**
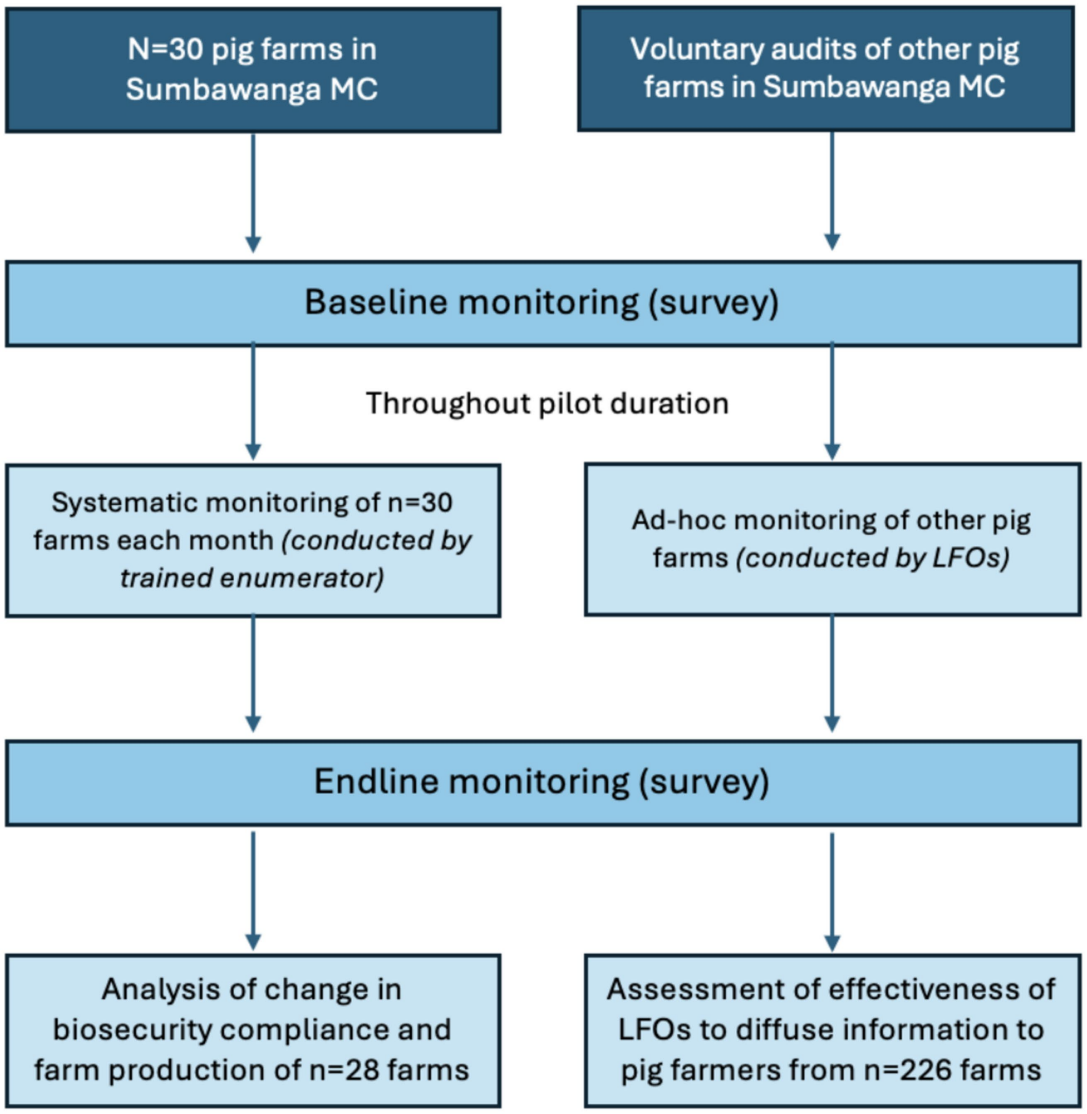
Study design used for data collection across pig farms in Sumbawanga MC.

In addition to the systematic monitoring, public and private LFOs were also requested to document observations from farm audits carried out on an ad-hoc basis (hereinafter referred to as ‘ad-hoc monitoring’). This ad-hoc monitoring was used as a proxy measure for assessing the effectiveness of LFOs to diffuse information about the biosecurity checklist to pig farmers. Those farm visits were entirely voluntary. LFOs were reimbursed 10 USD per month for the duration of the pilot period to cover for the costs of data usage. The study design for data collection is outlined in Figure 3.

#### 2.5.2 Qualitative

Three rounds of focus group discussions (FGDs) were conducted in Kiswahili with the LFOs between May and October 2024 coinciding with the start, midpoint, and end of the intervention. Each round focused on different themes: the first explored perceived challenges and initial reactions to the checklist; the second focused on evolving dynamics, including solutions and adaptations; and the third captured successes, relationship changes, and opportunities for scale-up. Each FGDs lasted on average for 60 min. Discussions were recorded through comprehensive field notes in the local language (Kiswahili), which were then translated into English by the bilingual facilitators. In addition to FGDs, the checklist survey from the systematic monitoring included some open-ended questions on aspects such as feed formulation and disease symptoms; although qualitative in form, these responses were categorised and are presented as quantitative summaries in Table 1.

**Table 1:** Sample characteristics at baseline of systematically monitored pig farms in Sumbawanga MC (*n* = 30).

Characteristic of farms/farmers	Categories	*n* (%)
Gender	Male	20 (66.7)
	Female	10 (33.3)
Level of education	Primary	11 (42.3)
	Secondary	5 (19.2)
	Diploma	7 (26.9)
	Bachelors	2 (7.7)
	Postgraduate	1 (3.8)
Herd size	1–5	6 (20.0)
	6–10	5 (16.7)
	11–20	11 (36.7)
	21–30	3 (10.0)
	31–40	3 (10.0)
	41–50	0 (0.0)
	>50	2 (6.7)
>12 months experience with pig farming	Yes	30 (100.0)
	No	0 (0.0)
Pig husbandry system	Confined (in pens) at all times	27 (90.0)
	Not confined (free-range)	3 (10.0)
Outbreak of ASF in the last 6 months before the pilot^*^	Yes	5 (16.7)
	No	25 (83.3)
Feed formulation^**^	Homemade feed^1^	30 (100.0)
	Commercial feed (pre-formulated or commercially formulated)	0 (0.0)
	Swill/kitchen scraps	9 (30.0)
	Other	0 (0.0)
Common signs of sickness as per farmers’ reports^**^	Diarrhoea	50 (52.6)
	Fever	2 (2.1)
	Lameness	16 (16.8)
	Skin (dermatological) condition	27 (28.4)
Farm location by ward	Chanji	4 (13.3)
	Kasense	4 (13.3)
	Kizwite	3 (10.0)
	Lwiche	2 (6.7)
	Majengo	3 (10.0)
	Mollo	3 (10.0)
	Momoka	1 (3.3)
	Ntendo	5 (16.7)
	Pito	5 (16.7)

* Based on anecdotal reporting, not laboratory confirmation of ASF infection.

** Multiple responses were selected, therefore total >100%.

1 Homemade feed is formulated using commonly available ingredients like broken maize, maize bran, sunflower meal, soybean meal and pig premixes.

### 2.6 Data analysis

#### 2.6.1 Quantitative

Data collected was exported from Kobo Collect and imported into Microsoft Excel. Descriptive analyses were conducted using Microsoft Excel (Version 2,411) by producing numerical summaries for continuous variables and frequency tables for categorical variables. Graphical figures (including histograms and boxplots) were also produced using Excel.

A baseline-and-endline approach was utilised, meaning the data collected at baseline during the first round of monitoring was compared with the data collected during the final phase of monitoring. Here, ‘baseline’ refers to the initial data collection intended for comparison after introducing the checklist. Where there were issues with data incompleteness, missing values were imputed with the value from the subsequent or previous monitoring phase for baseline and endline responses, respectively. This approach was selected to avoid significant deletion of data.

To investigate significant differences in checklist compliance scores and other parameters of interest (e.g., mortality, live weight, time spent implementing biosecurity and antimicrobial use) from baseline to endline, statistical tests were conducted using Excel and Epitools (26). Data were tested for normality using a visual assessment of a histogram and the Shapiro–Wilk test. After test assumptions were assessed, the Wilcoxon signed rank test was used to assess differences in the average biosecurity compliance scores between farms (from baseline to endline), and McNemar’s test was used to assess the difference in proportions between compliance to specific biosecurity practices at the baseline and endline of the intervention. The Wilcoxon signed rank test was used to compare the medians of the production parameters due to violations of the normality assumption.

#### 2.6.2 Qualitative

Qualitative data from the FGDs with the LFOs was analysed using an inductive and deductive approach. Initially, an inductive approach, based on thematic analysis (27), identified codes and themes using Microsoft Word independently by AA and KL. Subsequently, a deductive approach was employed where relevant identified themes were mapped onto the COM-B framework (18). This mapping aimed to complement and provide deeper contextual understanding to the quantitative findings derived from the TDF informed survey. Due to time and resource constraints, rapid qualitative research methods were utilised. Findings from the FGDs were analysed in conjunction with the quantitative results collected through systematic monitoring of farms to contextualise findings from the quantitative analysis and increase validity.

## 3 Results

### 3.1 Systematically monitored farms

A total of 30 farms were visited by the enumerator as part of the systematic monitoring of the pilot project (Figure 2). Two farms dropped out due to going out of business and hence only 28 complete responses were included for further quantitative analysis (i.e., beyond descriptive analysis). Farms scheduled for systematic monitoring were located in nine out of 19 wards (47.4%) in Sumbawanga MC. Twenty farmers were male (66.7%), 10 farmers (33.3%) were female and all farmers had over 12 months experience with pig farming. Majority of farmers received primary (42.3%) or secondary education (19.2%). The characteristics of the farms and farmers are presented in Table 1.

### 3.2 Baseline and endline analysis

#### 3.2.1 Overall biosecurity compliance

**Figure 4:**
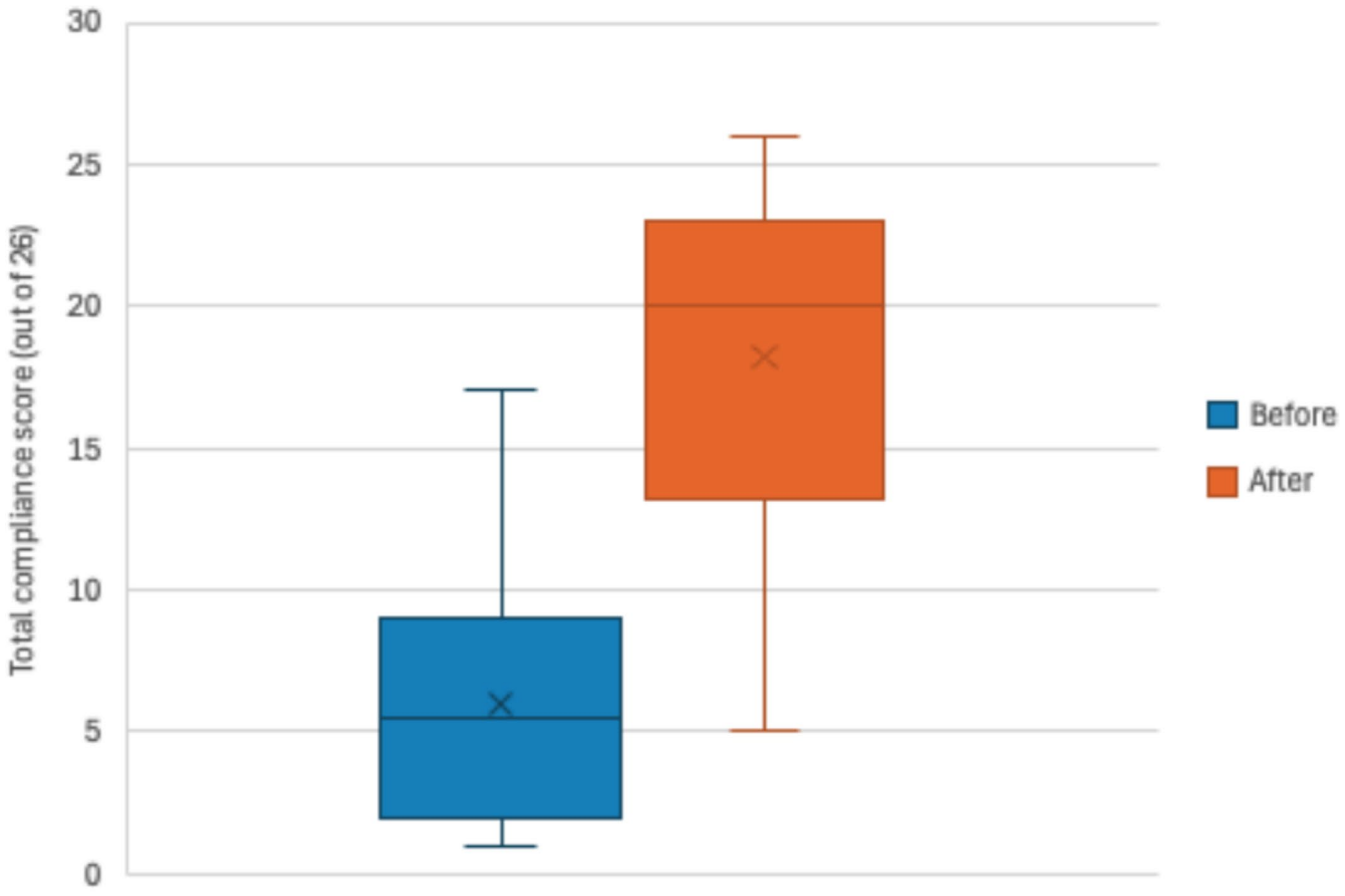
Total biosecurity compliance score (out of 26 practices) of pig farms at baseline (blue boxplot) and the endline (orange boxplot) of the pilot intervention (*n* = 30).

The biosecurity compliance score at baseline was on average 6.0 out of 26 practices or 23.1% (median: 5.5 practices, minimum: 1.0, maximum: 17.0) and after the pilot intervention was 18.2 out of 26 practices or 70.0% (median: 20.0, minimum: 5.0, maximum: 26.0) (Figure 4). The median compliance score was significantly higher after the pilot intervention (20.0 out of 26 practices or 76.9%) compared to baseline (median of 5.50 out of 26 practices 21.2%) (df = 27, *z* = 4.6, effect size = 0.87, *p* < 0.001).

**Figure 5:**
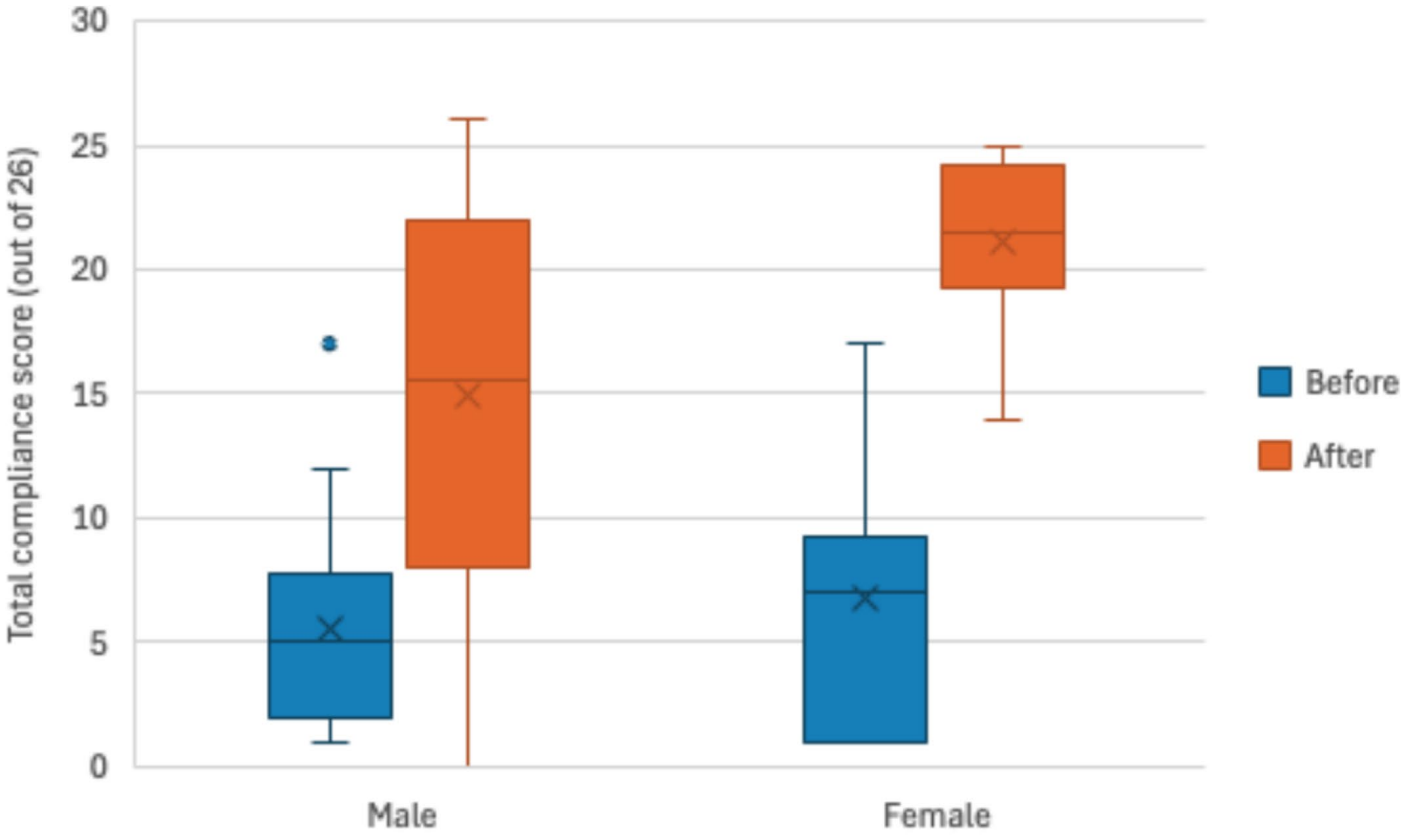
Total biosecurity compliance score (out of 26 practices) of pig farms by gender at baseline (blue boxplot) and after the endline (orange boxplot) of the pilot intervention (*n* = 30).

When looking at the change in compliance by gender of the farmer, at the baseline of the intervention, male farmers had an average compliance score of 5.6 out of 26 or 21.5% (median: 5.0, minimum: 1.0, maximum: 17.0) while female farmers had an average score of 6.8 out of 26 or 26.2% (median: 7.0, minimum: 1.0, maximum: 17.0). At the endline of the intervention, male farmers had an average compliance score of 16.6 out of 26 or 63.8% (median: 18.5, minimum: 5.0, maximum: 26.0) while female farmers had an average score of 21.1 out of 26 or 81.2% (median: 21.5, minimum: 8.0, maximum: 25.0). On average, female pig farmers adopted more practices than male farmers (Figure 5).

#### 3.2.2 Compliance by practice

All practices increased in compliance by the end of the pilot intervention (Table 2). The practices most frequently complied with at baseline were ‘keeping pigs confined at all times’ (*n* = 25, 89.3%); ‘segregating pigs by age groups’ (*n* = 19, 67.9%) and ‘no swill feeding’ (*n* = 20, 71.4%). The practices most frequently complied with at the end of the intervention were ‘keeping pigs confined at all times’; ‘no swill feeding’ and ‘segregating pigs by age groups’ (all *n* = 28, 100.0%).

**Table 2:** Compliance of farms to each good biosecurity practice included in the checklist at baseline and endline of the intervention (*n* = 28).

Good biosecurity practice	Baseline (before) *n* (%)	After *n* (%)	Δ% (95%CI)	*p*-value*
1. No visitors without permission	6 (21.4)	25 (89.3)	61.9 (50.6, 85.2)	**<0.001**
2. Pigs confined at all times	25 (89.3)	28 (100.0)	10.7 (0.7, 22.2)	0.248
3. Changing area before pen	1 (3.6)	14 (50.0)	46.4 (28.0, 64.9)	**<0.001**
4. Change boots and overcoat before pen	1 (3.6)	15 (53.6)	50.0 (31.5, 68.5)	**<0.001**
5. Segregate by age group	19 (67.9)	28 (100.0)	32.1 (14.8, 49.4)	**0.008**
6. Good housing structure	6 (21.4)	10 (35.7)	14.3 (1.32, 27.25)	0.134
7. Good housing conditions	8 (28.6)	15 (52.6)	25.0 (9.0, 41.0)	**0.023**
8. Access to clean water	14 (50.0)	27 (96.4)	46.4 (28.0, 64.9)	**<0.001**
9. Animals handled with care	12 (42.9)	23 (82.1)	39.3 (21.2, 57.4)	**0.003**
10. Clean farm area	6 (21.4)	23 (82.1)	60.7 (42.6, 78.8)	**<0.001**
11. Washing hands	8 (28.6)	16 (57.1)	28.6 (11.8, 45.3)	**0.013**
12. Cleaning and disinfection	1 (3.6)	17 (60.7)	57.1 (38.8, 75.5)	**<0.001**
13. No swill feeding	20 (71.4)	28 (100.0)	28.6 (11.8, 45.3)	**0.013**
14. Protected feed storage	2 (7.1)	17 (60.7)	53.6 (35.1, 72.0)	**<0.001**
15. Use clean farm equipment	3 (10.7)	23 (82.1)	71.4 (54.7, 88.2)	**<0.001**
16. Safe reproduction practices	12 (42.9)	13 (46.4)	3.6 (−3.3, 10.5)	1.0
17. Safe and prompt waste disposal	6 (21.4)	26 (92.9)	71.4 (54.7, 88.2)	**<0.001**
18. Good drainage on-farm	3 (10.7)	14 (50.0)	34.6 (16.3, 52.9)	**0.008**
19. Safe carcass disposal	4 (14.3)	27 (96.4)	82.1 (68.0, 96.3)	**<0.001**
20. Purchase disease free, healthy pigs	1 (3.6)	4 (14.3)	10.7 (−0.7, 22.2)	0.248
21. Isolate new and sick pigs	1 (3.6)	15 (53.6)	50.0 (31.5, 68.5)	**<0.001**
22. No movement or sale of sick pigs	0 (0.0)	19 (67.9)	67.9 (50.6, 85.2)	**<0.001**
23. Report sick pigs to veterinary services	7 (25.0)	23 (82.1)	57.1 (38.8, 75.5)	**<0.001**
24. Training on good husbandry	0 (0.0)	26 (92.9)	92.9 (83.3, 100.0)	**<0.001**
25. Use record keeping system	2 (7.1)	22 (78.6)	71.4 (54.7, 88.2)	**<0.001**
26. Prudent use of veterinary drugs	0 (0.0)	12 (42.9)	42.9 (24.5, 61.2)	**0.001**

*p-values <0.05 are bolded.

**Figure 6:**
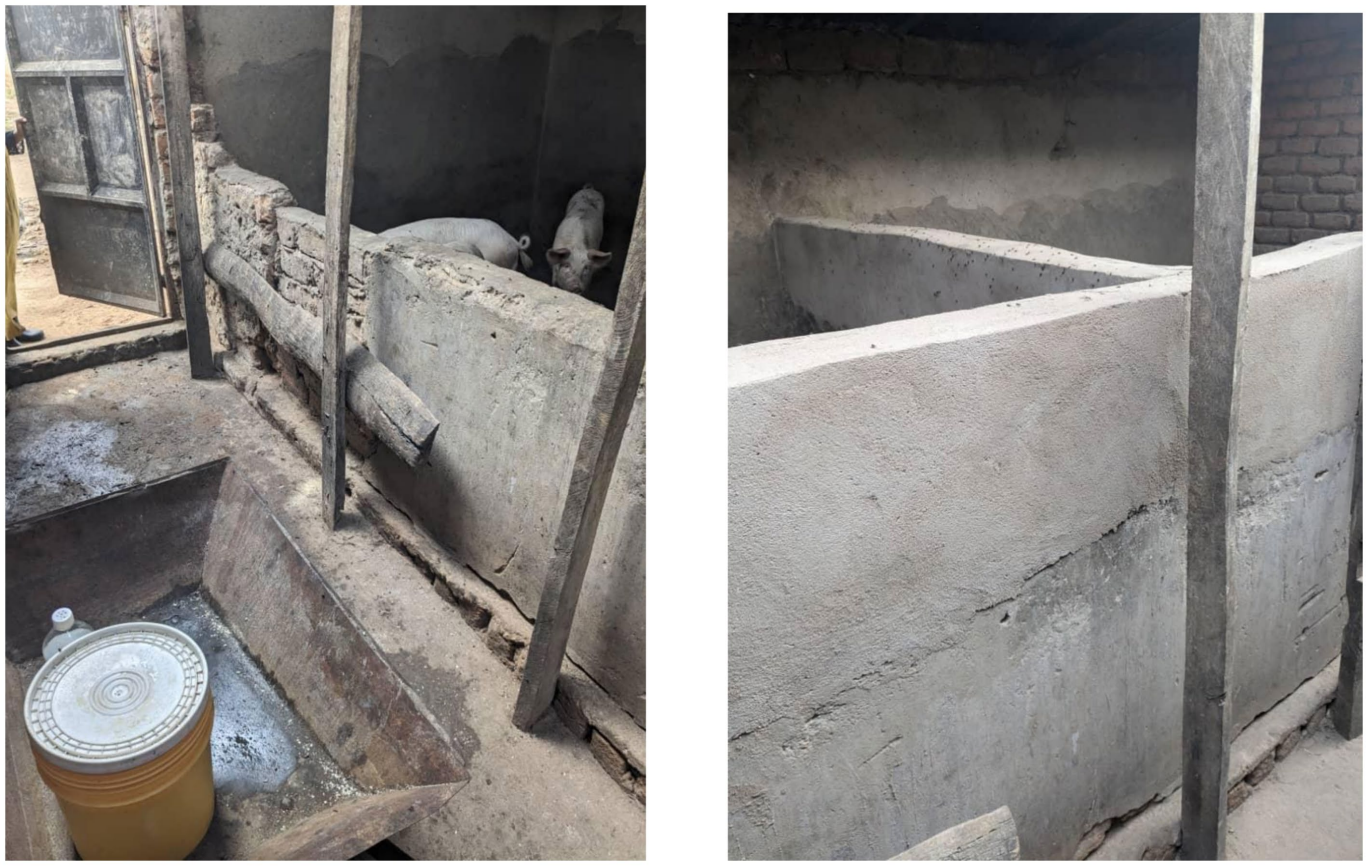
Example of improved housing structure with baseline image of pig farm pen (left) and after image of improved pig farm pen with raised wall height and structure (right). Improvements include segregation of pigs through installation of a new wall, fixing defects in existing and increasing height of walls to prevent contact of animals between pens.

The practices least complied with at baseline were ‘no movement or sale of sick pigs’; ‘training on good animal husbandry’ and ‘prudent use of veterinary drugs’ (*n* = 0). The practices least complied with after the intervention were ‘purchase of disease free, healthy pigs’ (*n* = 4, 14.3%); ‘good housing structure’ (*n* = 10, 35.7%) (see Figure 6) and ‘prudent use of veterinary drugs’ (*n* = 102, 42.9%).

The practices with the greatest improvement throughout the pilot duration were ‘training on good animal husbandry’ (92.9% improvement, *p* < 0.001), ‘safe carcass disposal’ (82.1% improvement, *p* < 0.001) and ‘safe and prompt waste disposal’ and ‘use of record keeping system’ (71.5% improvement, *p* < 0.001). On the other hand, practices with the least improvement were safe reproductive practices (3.6% improvement, *p* = 1.0), ‘keeping pigs confined at all times’ (10.7% improvement, *p* = 0.248) and purchase of disease free, healthy pigs (10.7% improvement, *p* = 0.248). Practices including ‘pigs being confined at all times’; ‘segregate by age group’ and ‘no swill feeding’ all improved to 100% compliance by the end of the pilot intervention.

### 3.3 Production-related parameters

The findings of production-related parameters measured at the baseline and endline of the pilot intervention are summarised in Table 3.

**Table 3:** Characteristics of participating pig farms monitored (at baseline and endline) as part of the pilot intervention in Sumbawanga MC (*n* = 28).

Characteristic	Baseline		Endline		*p*-value
	Mean (SD)	Median [Min, Max]	Mean (SD)	Median [Min, Max]	
Total biosecurity compliance score (out of 26)	6.0 (4.3)	5.5 [1.0, 17.0]	18.2 (6.4)	20.0 [5.0, 26.0]	<0.001
Number of sows per herd	3.1 (3.3)	2.0 [0.0, 14.0]	3.6 (4.3)	2.5 [0.0, 22.0]	0.73
Litter size	6.9 (4.1)	8.0 [0.0, 12.0]	7.0 (1.6)	7.0 [5.0, 10.0]	0.69
Herd size	18.0 (19.6)	13.0 [1.0, 91.0]	18.7 (23.4)	12.0 [1.0, 125.0]	0.93
Pre-weaning mortality (%)	33.6 (18.2)	28.6 [12.5, 75.0]	27.6 (8.6)	25.0 [14.3, 42.9]	0.21
Time spent implementing biosecurity per sow (minutes)	13.2 (12.2)	7.8 [2.0, 60.0]	28.0 (24.3)	18.6 [6.4, 120.0]	0.0037
Proportion of herd treated with antimicrobials per month (%)	58.4 (43.7)	68.1 [0.0, 100.0]	37.2 (42.8)	6.7 [0.0, 100.0]	0.08
Cost of AMU per month per sow in TZS (USD)	7,506.5/2.8 (6,863.3/2.6)	7,500.0/2.8 [0.0/0.0, 27,500.0/10.2]	5,035.9/1.9 (6,574.2/2.4)	3,333.3/1.2 [0.0/0.0, 30,000.0/11.1]	0.085

#### 3.3.1 Production parameters

The number of sows per herd increased from an average of 3.1–3.6 sows (median increased from 2.0 to 2.5) and the average litter size increased from an average of 6.9–7.0 piglets (median decreased from 8.0 to 7.0 piglets). The herd size increased from an average 18.0–18.7 heads (median reduced from 13.0 to 12.0), while the average pre-weaning mortality had decreased from 33.6 to 27.6% (median decreased from 28.6 to 25.0%). None of these differences were found to be statistically significant (Table 3). Some variables such as litter size have a minimum value of 0, indicating that the farm did not have any pigs at the time of the visit, which could be attributed to factors such as a recent sale or a disease outbreak. Post-weaning mortality, average daily gain and age when sold for slaughter were also assessed, however, due to incompleteness of data and inadequate counts, no statistical testing was carried out.

#### 3.3.2 Time spent implementing biosecurity

The (self-reported) time spent implementing biosecurity per sow and day increased from an average of 13.2–28.0 min or 71.8% by the end of the intervention (median increased from 7.8 to 18.6) (Table 3).

#### 3.3.3 Antimicrobial use (AMU)

The average proportion of the herd treated with antimicrobials per month decreased from 58.4 to 37.2% animals by the end of the intervention (median reduced from 68.1 to 6.7%). The cost of AMU per sow (per month) also reduced from an average of 7,506.5 TZS (2.8 USD) to 5,035.9 TZS (1.8 USD) (median of 7,500 TZS (2.8 USD) to 3,333.3 TZS (1.2 USD)), i.e., a 57% reduction. However, both of these differences were not found to be statistically significant (Table 3).

### 3.4 Ad-hoc monitored farms

A total of 226 pig farms were visited (audited) as part of the ad-hoc monitoring conducted by LFOs in Sumbawanga MC from May to October 2024. The majority of farmers were male (61.1%) and most of the farms had a herd size of 1–5 pigs (*n* = 100, 44.2%) or 6–10 pigs (*n* = 56, 24.8%) while only four farms (1.8%) had >50 pigs in the herd.

**Figure 7:**
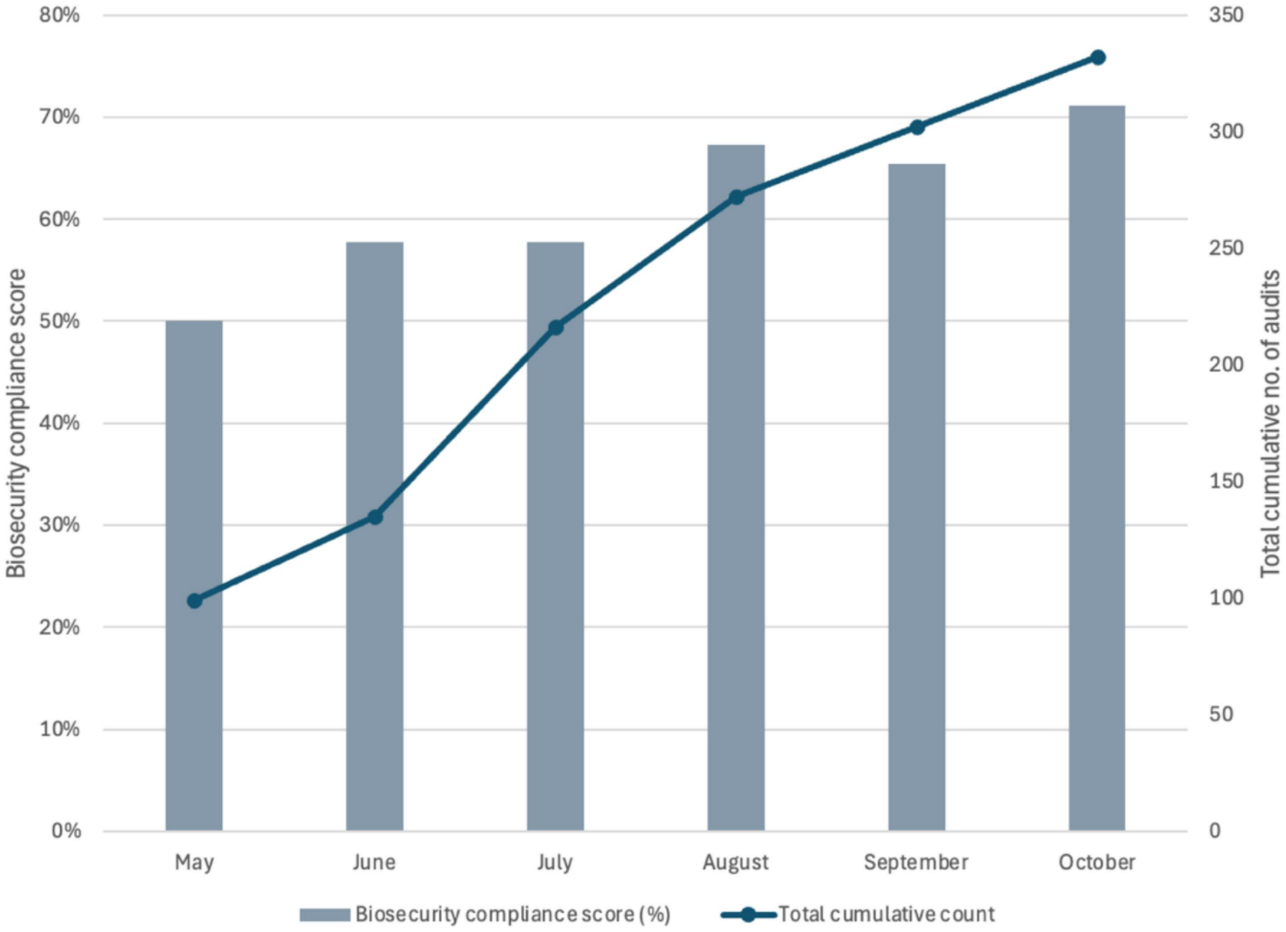
Bar chart displaying biosecurity compliance score (%) on primary y-axis and total number of audits completed (including revisits) through ad-hoc monitoring by livestock field officers in Sumbawanga MC by month (secondary y-axis).

Occasionally, farms were audited more than once by LFOs—a total of 69 farms (30.5%) were audited more than once and up to six times throughout the pilot duration. The average biosecurity compliance score at baseline was 13.0 out of 26 practices or 50.0% (median: 15.0, SD: 5.3, minimum: 1.0 and maximum: 26.0) and at the end of the intervention was 17.9 out of 26 practices or 69% (median: 18.4, SD: 5.7, minimum: 4.0, maximum: 26.0). The number of pig farms audited per month throughout the pilot duration remained consistent, with small increases in the number of farms audited in August and October (Figure 7). However, it should be noted that the cumulative number of audits depicted in Figure 7 includes revisits conducted on farms already visited by LFOs previously. The farms monitored on an ad-hoc basis by LFOs also showed a similar level of improvement as the systematically monitored farms. Additionally, a steady rate of submissions of auditing data was received throughout the pilot duration (Figure 7).

### 3.5 Behavioural factors

#### 3.5.1 Enabling and disabling factors associated with implementing good biosecurity practices

A central aim was to understand the behavioural determinants influencing the uptake of biosecurity practices. This was approached through complementary methods during the final phase of monitoring (i.e., at the end of the intervention). Farmers were asked about community and individual level perspectives on barriers and enablers to implement the checklist. Using a survey informed by the TDF (17) (see Table 4). Further analysis has been provided in the Discussion section.

**Table 4:** Perceived influences on the implementation of biosecurity measures: farmer-reported barriers and facilitators assessed at intervention endline.

Good practice	Enabling factors	Disabling factors
1. No visitors without permission	have some knowledge about why the practice is necessary somewhat confident in ability but very motivated to continue or begin implementing feel rewarded when the practice is implemented (by limiting entry of disease) feel very positive and optimistic that the practice will benefit farming business	sometimes forget about practice; only done out of habit sometimes no resources available to implement practice (e.g., gates, fencing) very uncommon practice amongst pig farmers in the community only somewhat concerned when the practice is not implemented
2. Pigs confined at all times	have some knowledge about why the practice is necessary and often done out of habit. Very motivated and very confident in ability to implement feel rewarded when the practice is implemented (by protecting pigs against spreading disease) feel very concerned when the practice is not implemented feel very positive and optimistic that the practice will benefit farming business	sometimes forget about practice limited resources available to implement very uncommon practice amongst pig farmers in the community limited resources to build improved housing structures for pigs people whose opinions farmers value are neutral or indifferent about the practice
3. Changing area before pen	have extensive knowledge about why the practice is necessary and very confident in ability to implement. Never or rarely forget to implement people whose opinions farmers value strongly encourage the practice very motivated and feel rewarded implementing the practice (by reducing cost of treatments, preventing deaths, improving safety of pigs) feel very concerned when the practice is not implemented	limited resources, time and technical guidance available to implement the practice (build changing area) very uncommon practice amongst pig farmers in the community
4. Change boots and overcoat before pen	have some knowledge about why the practice is necessary and somewhat confident in ability to implement somewhat motivated and feel rewarded when the practice is implemented (because farmers will protect their animals and not be responsible for spreading diseases in their farm) feel very concerned when the practice is not implemented	sometimes forget to implement the practice and only done sometimes out of habit no resources available to implement the practice (i.e., buy an overcoat) people whose opinions farmers value are neutral or indifferent about the practice it is an uncommon practice amongst pig farmers in the community
5. Segregate by age group	have some knowledge about why the practice is necessary and it is often done out of habit. Feel very confident and motivated in ability to implement people whose opinions farmers value somewhat support implementing this practice feel rewarded and somewhat optimistic when the practice is implemented (since the farmers is protecting their pigs, which will lead to a more profitable farm)	sometimes forget to implement the practice it is an uncommon practice amongst pig farmers in the community
6. Good housing structure	have extensive knowledge about why the practice is necessary feel very confident and motivated in ability to implement feel positive when it is implemented and very concerned when it is not implemented feel rewarded when implemented (by providing safety to animals and money that has been invested) strongly encouraged by people whose opinions farmers value	requires money and resourcing to have good housing structure
7. Good housing conditions	have some knowledge about why the practice is necessary and somewhat confident in ability to implement feel rewarded when implementing the practice it is a common practice in the community and very motivated to implement	limited resources available to implement feel as though people whose opinions farmers value are neutral or indifferent about the practice
8. Access to clean water	have some knowledge about why the practice is necessary and feel confident in ability to implement. Often it is done out of habit have resources available to implement this practice; it is strongly encouraged and a common practice in the community feel rewarded when implementing the practice (when pigs’ water intake is high, and they are protected from water-borne disease) feel very concerned when the practice is not implemented correctly	no challenges reported
9. Animals handled with care	have some knowledge about why the practice is necessary and feel confident in ability to implement have resources available to implement this practice	the practice is only somewhat supported in the community feel only somewhat motivated to begin or continue implementing this practice and it is done sometimes out of practice do not feel rewarded when implementing this practice
10. Clean farm area	have some knowledge about why the practice is necessary it is strongly encouraged and a very common practice in the community. Often the practice is done out of habit very motivated and feel rewarded when the practice is implemented (since the farm area is clean and the pigs are therefore clean and healthy)	sometimes forget to implement the practice have limited resources available to implement the practice—it requires money to buy items to help with cleaning
11. Washing hands	have extensive knowledge about why the practice is necessary never forget about this practice and often done out of habit now feel motivated and rewarded to implement practice (as it will save on money to treat pigs with less sickness and potentially make the farm more profitable)	can be challenging to have financial resources to purchase or build handwashing station uncommon practice amongst pig farmers in the community
12. Cleaning and disinfection	have some knowledge about why the practice is necessary feel very motivated to begin or continue following the practice and feel rewarded (since it will contribute to having healthier animals or avoiding disease which will make the farm more profitable)	only somewhat confident in ability to implement the practice sometimes forget to implement the practice and sometimes done out of habit have limited resources to implement the practice people whose opinions farmers value are neutral or indifferent about the practice and it is uncommon in the community
13. No swill feeding	have extensive knowledge about why the practice is necessary feel confident in ability to implement and is always done out of habit strongly encouraged by people in the community and is common feel rewarded (since pigs will be healthier)	commercial or pre-formulated feeds can be expensive and so sometimes farmers may opt to feed kitchen scraps/leftovers
14. Protected feed storage	have some knowledge about why the practice is necessary feel motivated and rewarded when implemented (since it will mean diseases aren’t spread to pigs via feed)	sometimes forget to implement the practice and somewhat supported by others in the community whose opinion farmers value uncommon practice amongst pig farmers in the community and only sometimes done out of habit can be high cost associated with having to build protected area for feed
15. Use clean farm equipment	have some knowledge about why the practice is necessary feel motivated and rewarded when the practice is implemented (since farmers will prevent the entry and spread of disease within their pig farm)	only somewhat confident in ability to implement the practice sometimes forget to implement the practice have limited resources to implement the practice only somewhat concerned when the practice is not implemented
16. Safe reproduction practices	have good knowledge about why the practice is necessary feel very confident and motivated in ability to implement resources are available to implement the practice feel rewarded when the practice is followed (since diseases through breeding can be avoided but can still access/keep the breeds that the farmer wants)	people whose opinions farmers value are neutral or indifferent about the practice and it is very uncommon in the community
17. Safe and prompt waste disposal	have extensive knowledge about why the practice is necessary feel very confident in ability to implement, motivated and as though all the resources are available the practice is strongly encouraged by people whose opinions farmers value it is a very common practice in the community and done out of habit	challenges include area for solid waste disposal being is far away from pig housing and lacking equipment to transport waste (increasing manual labour required for this practice)
18. Good drainage on-farm	have some knowledge about why the practice is necessary and feel somewhat confident in ability to implement very motivated to implement and feel rewarded (as it contributes to cleanliness of the farm)	sometimes forget to implement the practice and somewhat supported by others in the community whose opinion farmers value limited resources available to implement the practice only sometimes done out of habit
19. Safe carcass disposal	have some knowledge about why the practice is necessary and feel very confident in ability to implement very motivated and feel rewarded when implementing the practice often done out of habit	implementing the practice is somewhat supported by others in the community whose opinion farmers value challenges include space on farm for appropriate carcass disposal
20. Purchase disease free, healthy pigs	have some knowledge about why the practice is necessary	only somewhat confident in ability to implement the practice have no resources available to support implementing the practice people whose opinions farmers value are neutral or indifferent about the practice and it is very uncommon in the community not at all concerned when the practice is not implemented or overlooked practice is never done out of habit given the costs involved
21. Isolate new and sick pigs	strongly encouraged by others in the community whose opinion farmers value motivated to implement the practice and feel rewarded when it is implemented (since it will maintain healthy animals, prevent the spread of disease and increase productivity)	have little knowledge about why the practice is necessary only somewhat confident in ability to implement the practice have limited resources to implement the practice and often difficult in small farm areas it is an uncommon practice amongst pig farmers in the community and rarely done out of habit
22. No movement or sale of sick pigs	feel somewhat motivated to implement the practice the practice is somewhat commonly done believe that the practice is somewhat important and they are somewhat optimistic that this practice would improve farming business	have little knowledge about why the practice is necessary and do not feel confident in ability to implement the practice is often forgotten about and never done out of habit do not feel rewarded when the practice is adopted the practice is somewhat discouraged by people in the community whose opinions farmers value farmers feel as though they will lose money if they do not sell animals challenges to implementing the practice are no compensation schemes for sick/dead animals
23. Report sick pigs to veterinary services	have some knowledge about why the practice is necessary feel motivated to implement the practice very common practice amongst pig farmers in the community but only done sometimes out of practice	only somewhat confident in ability to implement the practice sometimes forget to implement the practice and report and only somewhat supported by others in the community whose opinions farmers value do not feel rewarded when the practice is implemented because there is difficulty in getting LFOs to visit (poor availability) and trust issues to effectively treat animals
24. Training on good husbandry	have extensive knowledge about why the practice is necessary feel positive and very motivated to implement the practice since it increases their knowledge and awareness on issues related to farming	limited resources or opportunities to implement this practice (there is an issue to be involved in trainings frequently)
25. Use record keeping system	have some knowledge about why the practice is necessary feel confident in ability to implement, motivated and have the resources available strongly encouraged by others in the community and somewhat common feed rewarded when the practice is implemented (since it shows how their farm and business is growing)	can be challenging and demanding but feasible with guidance and training
26. Prudent use of veterinary drugs	feel very motivated and rewarded when the practice is implemented (since limiting unnecessary drug use saves on expenditure and improves animal health) feel positive when the practice is followed	have little knowledge about why the practice is necessary and only somewhat confident in ability the practice sometimes forget to implement the practice and it is rarely done out of habit people whose opinions farmers value are neutral or indifferent about the practice and it is very uncommon in the community limited resources to build a proper place for storage of vet drugs difficult to break out of habit to reach for vet drugs whenever something is wrong with animals

Behavioural determinants extracted during the final monitoring phase were further complemented and contextualised by conducting FGDs, asking LFOs about barriers to implement the checklist or to perform their role as agents of change to improve biosecurity amongst pig farmers. The themes and codes extracted from the FGD conducted are presented in Tables 5–7.

**Table 5:** Themes and codes from FGDs conducted with LFOs during the training delivered in May 2024 in Sumbawanga MC.

Discussion question: *What was learnt about implementing the checklist in the first four weeks of the pilot?*			
Themes	Theme 1: Awareness of farmers	Theme 2: Farming systems	Theme 3: Distrust of the checklist/pilot
Codes	Awareness of ASF Farmers unaware of how checklist prevents pig diseases Poor economic status hindering willingness	Pig farming systems not aligned with checklist recommendation Existing farming systems are porous and not secure	False information about pig disease and deaths Inconsistent commitment to visits from LFOs

Discussion question: *Were there any challenges or barriers to implementing the checklist?*			
Themes	Theme 1: Lack of interest in the checklist/pilot	Theme 2: Distrust of the LFOs; checklist/pilot	Theme 3: Factors impacting capacity
Codes	Farm owner cannot be located Farmers not readily available Not willing to give up time Farmers not interested Lack of interest to submit information for audit Farmers feel like time wasted	Unwillingness to answer questions False information provided	Lack of transport for farm visits Lack of internet to submit audit data Persons looking after pigs have poor knowledge to support with checklist audit

Discussion question: *Were there any successful experiences? What did farmers respond best to?*		
Themes	Theme 1: Level of awareness and trust	Theme 2: Readiness to adopt measures
Codes	Good level of awareness Ready to listen to LFOs	Readiness to adopt measures Started keeping records Improved cleaning of pens and farm environment Use of protective gear Improved drainage

**Table 6:** Themes and codes from FGDs conducted with LFOs during the training delivered in June 2024 in Sumbawanga MC.

Discussion question: *Is there any form of remuneration (monetary and non-monetary) made between LFO and farmers? Under which conditions are livestock field officers usually paid for extension services?*			
Theme	Theme 1: Types of remuneration between farmer and LFO	Theme 2: Remuneration for public servants	Theme 3: Remuneration for private business owners
Codes	Remuneration based on conditions Monetary remuneration for vet drug administration and procedures (by both public and private sector) Non-monetary remuneration often in the form of gifts (chicken, eggs, groundnuts, maize)	Unable to charge transport fees (despite demand for) Advisory services provided free-of-charge Charge no consultation fee	Private employees set costs/fees based on item cost Charge no consultation fee Transportation and drug costs charged to farmer

Discussion question: *Why is remuneration for extension services provided in some instances and not others?*			
Themes	Theme 1: Factors related to the farmer	Theme 2: Factors related to the extension service provider	Theme 3: External factors
Codes	Willingness of the farmer to provide remuneration Level of hardship encountered Demand of service Level of expertise needed through services	Level of technical knowledge Ability to provide services remotely (e.g., telephone consultation) Demand of service	Recovering financial costs of drugs and equipment

**Table 7:** Themes and codes from FGDs conducted with LFOs during the midterm meeting in August 2024 in Sumbawanga MC.

Discussion question: *What are some of the challenges that you have encountered in the pilot so far?*			
Theme	Theme 1: Inconsistent sensitisation and interest	Theme 2: Factors impacting capacity	Theme 3: Solutions to challenges
Codes	Inconvenience when farm workers are helping with audit (“starting from zero”) Farmers only interested in the checklist when ASF occurring during the rainy season Farmers with only a few animals not interested in long-term improvements Farmers set in traditional way of doing	Lack of transport allowance for farm visits Lack of internet to submit audit data Farmers have limited resources to implement certain practices	Simple flyers to sensitise Educate more widely Commitment from local government through allocation of regular budget Creativity or innovative solutions to convince those last farmers

Discussion question: *How has your relationship with farmers changed during the pilot? (How have you worked in partnership with farmers?)*		
Themes	Theme 1: Improved trust	Theme 2: Unintended benefits
Codes	Checklist framed as a solution from the government, produced after listening to farmers Trust improved after seeing benefits Improved recognition of LFOs Improved communication with farmers following training Use of gender-sensitive approaches following training	Gift giving from farmers and some payments Word of mouth leading to more farmers requesting services Checklist can be adapted for use in other livestock systems Usually, only cattle and poultry farmers working with LFOs, now also pig farmers Improved confidence amongst LFOs

Themes, which emerged during the FGD in terms of challenges and barriers included a general lack of interest from farmers in the checklist approach to improve biosecurity; distrust of working with LFOs and the approach and factors that impacted capacity, such as lack of transport for LFOs to conduct farm audits (Table 5). FGDs were repeated at the midpoint of the pilot, where challenges related to capacity were reiterated. However, at this point, LFOs were able to offer solutions to the problems like simple flyers about the checklist or education (sensitisation) of the entire family or farm staff to enhance understanding. Despite the efforts, it was acknowledged that some capacity issues would only be resolved through commitment of additional resource allocation by the local government (Table 7).

During the pilot design phase, remuneration from farmers had been proposed as a possible solution to sustainably overcome the capacity issues faced by LFOs (as opposed to resourcing directly provided through the project). This could be monetary or non-monetary remuneration. However, the discussions elucidated that public LFOs were unable to charge farmers for transport fees or provision of technical, extension advice. Remuneration can only be provided for provision of veterinary drugs or performing procedures and therefore, was not deemed to be a suitable solution for public LFOs in the long-term.

Conversely, themes highlighting successful factors and experiences included the level of awareness of biosecurity amongst farmers and readiness of some farmers to listen and work with LFOs. Opportunities included connecting LFOs to new farmers to provide services and collaboration between LFOs to co-develop solutions for farmers (Table 7). Despite the initial lack of trust, relationships between LFOs and farmers were described to have transformed throughout the pilot duration, highlighting another success of the pilot intervention. LFOs were trained on effective communication and using gender-sensitive approaches, which were reported to be factors contributing towards working more effectively in partnership with farmers. When LFOs advised farmers that the checklist had been proposed as a solution in response to hearing the problems of farmers, this improved trust and strengthened relationships.

## 4 Discussion

### 4.1 Compliance to biosecurity

This pilot intervention found there is utility of a co-created and progressive checklist approach—where farmers are encouraged to prioritise practices to implement based on risks on farms and feasibility, rather than adopt several practices immediately—as evidenced by the significant improvement in the adoption of biosecurity measures at the farm level by the end of the intervention. The baseline biosecurity compliance was low at 23%, indicating substantial room for improvement. This finding aligns with a recent study conducted in the neighbouring region of Mbeya, Tanzania, where biosecurity was assessed on pig farms using a 25-item biosecurity checklist and the mean score for premises evaluated was 29% (3). The poor baseline biosecurity could reflect the low-input, low-output pig production systems in Tanzania (28); farmers’ unwillingness to invest; limited understanding of how to encourage farmers to change and adopt measures and/or a lack of enforcement and/or technical knowledge from LFOs.

The approach used in this pilot intervention is tailored to the local context and has been validated by local stakeholders to ensure it is practical, feasible and affordable. Other approaches, like farmer field schools (FFS), which are also based on a bottom-up participatory approach (29) have reported similar findings, in that FFS participants also reported significantly higher infection, prevention and control (i.e., basic hygiene or biosecurity measures) scores compared with non-FFS respondents (30).

Female farmers were found to adopt a greater number of practices throughout the pilot. Female farmers in Sumbawanga MC have a prominent role in pig farming—many are sole keepers of pigs while others are joint owners with men in the household (31). Training or sensitisation around biosecurity should, therefore, explicitly target both men and women in the households to ensure equal participation and benefit from income-earning opportunities. To facilitate this, LFOs were trained in using gender-sensitive approaches when providing extension services to ensure that female farmers feel comfortable interacting with animal health services, who are responsible for creating opportunities for women to reduce their exposure risk (as female farmers are more often responsible for day to day tasks like, feeding, cleaning and looking after sick pigs which puts them at higher risks of being exposed to or inadvertently transmitting disease) and empower them to improve their pig enterprises and profitability.

**Figure 8:**
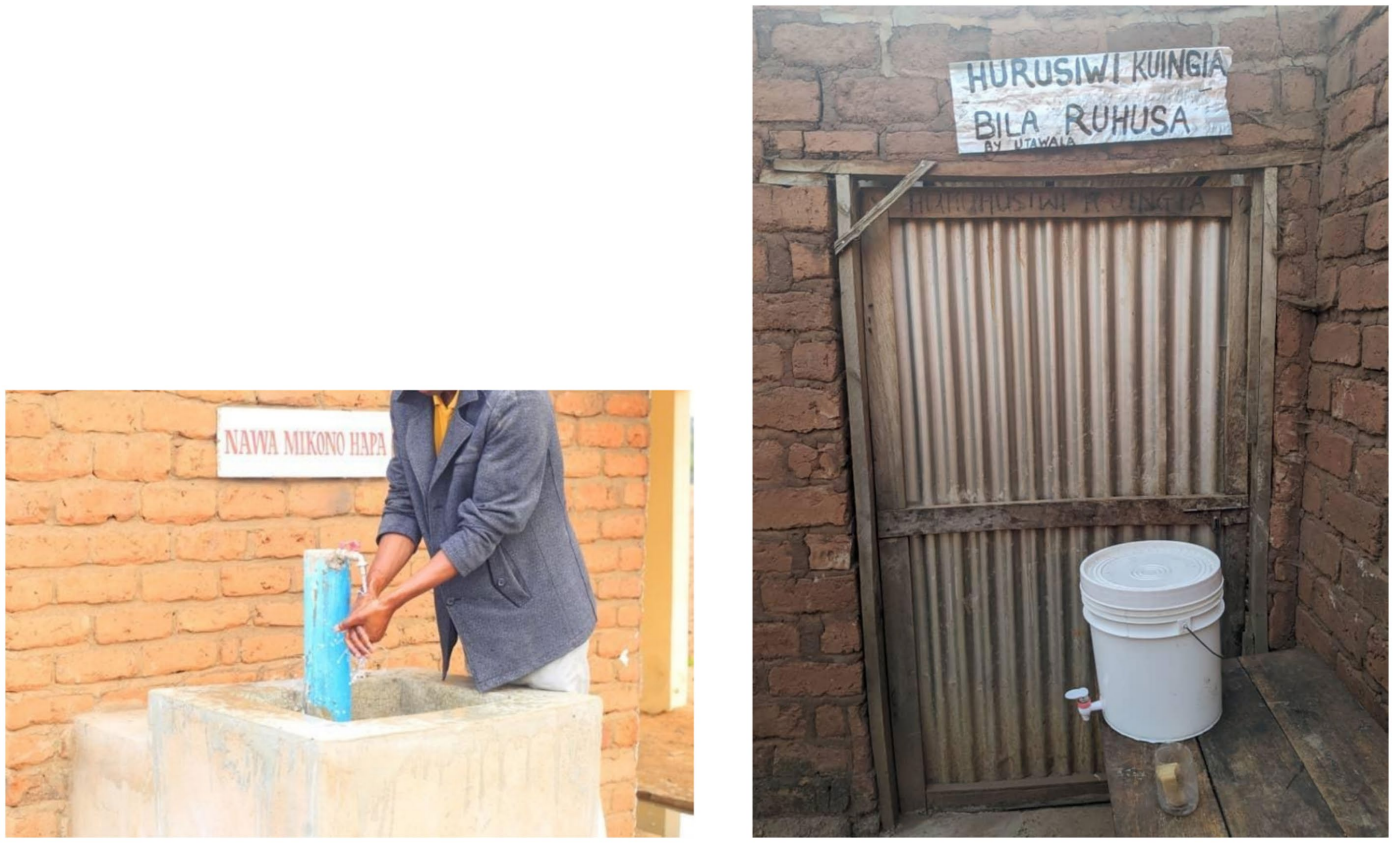
Examples of different types of handwashing stations implemented within participating farms. Both images also include signs with text in Kiswahili, which indicates to restrict entry of visitors onto the farm.

The findings of this study show that different changes in behaviour do not necessarily have the same benefits/enablers and/or barriers/disablers and as such need different approaches and interventions to result in implementation. Despite increased knowledge of biosecurity practices, data indicate that uptake remains low for certain practices, such as ‘good housing structure’, and ‘purchase of disease free, healthy pigs’. Our findings are consistent with other studies investigating biosecurity uptake (8, 32). Key barriers identified through the TDF-informed assessment to implementation of many practices in the checklist, related to the Opportunity component of COM-B, specifically Environmental Context and Resources (e.g., limited finances for housing improvements) and Social Influences (e.g., lack of supportive community norms or encouragement from peers for novel practices). Additionally, challenges related to Psychological Capability, such as Behavioural Regulation or Memory, Attention and Decision Processes, were evident in difficulties establishing habits or remembering specific steps for less routine practices. To address these challenges, in the pilot intervention, there was a great importance paid to practical solutions feasible to implement and progressively improve with existing resources or options to avoid prohibitive costs preventing farmers from investing in practices. Examples include use of jerry cans as handwashing stations (that might be upgraded to proper taps later as illustrated in Figure 8); use of detergent or other cost-effective alternatives like pure vinegar or whitewash (rather than cost-prohibitive disinfectants) and encouraging safe reproductive practices for farmers that cannot afford their own breeding boar and continue to borrow boars from neighbouring farmers. However, in the case of washing hands or safe reproductive practices, a substantial improvement in compliance wasn’t seen, which may simply indicate that a longer time frame is required to see changes.

However, this last practice on safe reproduction shows that despite efforts and focus on progressive improvements, there remain persistent challenges beyond farmers’ control that can only be marginally improved without a more targeted structural support, such as availability of government-approved health breeding stock. Similarly, practices such as ‘no movement or sale of sick pigs’ faced Motivation barriers, particularly related to Reinforcement (farmers reported not feeling rewarded) (unlike for other practices, where this was frequently found to be an enabling factor) and negative Beliefs about Consequences (fear of financial loss) Addressing this likely requires intervention beyond individual farmer motivation, potentially through longer-term solutions like public-private-partnerships to fund compensation mechanisms that are implemented by the competent authority or insurance systems may be considered.

Sensitisation events were held to raise awareness so that practices were no longer seen as uncommon amongst farmers in the community, in addition to providing a space where farmers learn about behaviours of other farmers in their peer group and a sense of community is strengthened (33). It is well recognised that practices like the purchase of disease-free and healthy pigs are challenging, and to encourage adoption, partnerships with the private sector, for instance, the Tanzania Association of Pig Farmers (TAPIFA) could be explored during the scale up phase. TAPIFA is already responsible for sourcing pigs for its members and extending access of memberships to small-scale farmers may provide an opportunity or incentive to overcome this challenge. Approaches that take into consideration individuals’ preparedness for change as well as motivations for behaviours may provide a framework or catalyst for tangible change in biosecurity (15). Recognition of farmers that improve practices substantially can facilitate the identification of champions or ambassadors whose example can be used to foster changes among their peers.

Both farmers and public extension officers have transformed into agents of change in this pilot intervention through being empowered to contribute towards innovative solutions to improve biosecurity. In particular, LFOs have created an intent in farmers to change, adopt good practices and diffuse information while establishing information-exchange relationships between themselves to share experiences and co-create solutions. In this way, LFOs are key to the success of such interventions and are likely to also function as scaling up agents on the ground in the next phase of the project.

### 4.2 Association between biosecurity, production and antimicrobial use

In terms of the impact on production, the study faced challenges to show a significant reduction in several parameters including pre-weaning and post-weaning mortality, as well as an impact on average daily gain after the intervention due to limited data and short timeframe of the study. However, the proportion of the herd treated with antimicrobials and the cost of antimicrobials also reduced by the end of the intervention (showing reductions of over 50% in both parameters). Given there was no statistically significant difference between baseline and endline, it is important to note that no inferential statements can be made about the effect of biosecurity on mortality rates or antimicrobial use and expenditure in this study and caution is recommended when drawing conclusions. This may be due to the study being underpowered to detect smaller effect sizes.

However, these findings do not indicate that there is an absence of an effect. It is likely that this is due to a type II error resulting in non-significance due to the study being underpowered, with a small sample size, which was a consequence of logistical challenges related to data collection when carrying out field studies in lower- and middle-income settings.

Few studies have been conducted to illustrate the association between biosecurity, production parameters and antimicrobial use in pig herds, and majority of these studies have been carried out in farrow-to-finisher production systems within high-income contexts like the European Union (34–36). This means these findings are difficult to extrapolate to value chains in low-resource or lower- and middle-income settings where smallholder or small- to medium-scale farming systems predominate. It is essential to prioritise collaborations (for instance, through training LFOs to become enumerators) to improve the quantity and quality of data collected, thereby avoiding limitations in terms of data collected and inconclusive findings.

### 4.3 Sustainability

Sustainability remains a challenge for many community-level health interventions. Sustainability is defined as “the extent to which an intervention can deliver its intended benefits over an extended period after external support is terminated” (37). The approach used for this intervention was novel for development agencies in that no funding or resourcing was provided to directly support farmers to improve biosecurity on their farms, but instead, funding was only provided for capacity development through training (of LFOs as our scaling agents) and activities, such as sensitisation and recognition events. This was primarily to ensure farmers understood their individual responsibility to invest and improve biosecurity on their farms and to ensure sustainability beyond the pilot duration. This was achieved through using a specifically designed participatory, co-creation approach (22) where the local stakeholders are empowered to be the innovators of solutions.

Sustainability relies on the local government absorbing responsibility for implementation going forward, i.e., to continue at the local level and scale up, for instance, into surrounding local government administrations. The strong and steady support from LFOs doing voluntary farm visits as part of the ‘ad-hoc’ monitoring is a promising sign of commitment showing that the improvements are not limited to the systematically monitored farms. In this regard, another positive sign is the fact that the local government in Sumbawanga MC has committed to integrate biosecurity audits into the regular work of LFOs after the pilot’s completion, and regular budget allocation towards fuel allowance for LFOs to perform farm audits. This commitment came about after the data collected by LFOs during the pilot project were presented to the local government and it has been recognised that fuel allowance is necessary to sustain this level of extension service provision. This highlights the importance of working directly with and involving all stakeholders, including the local government. An alternative solution proposed to this challenge was remuneration for LFOs paid by the farmers. An example of the practical application of this solution was through organisation of farmers associations, where a proportion of member fees would be allocated to LFOs to ensure regularity of extension service provision, however, this was not implemented as part of the pilot intervention. Although this was suggested during the project design phase by farmers, FGDs conducted with LFOs revealed that public extension officers provide advisory services free-of-charge and monetary remuneration can only be provided for veterinary drug administration and procedures, rendering this solution unfeasible in the context of Tanzania, unless a cost-recovery policy reform is implemented.

It is important to note that a significant increase in compliance resulted following a sensitisation event, where farmers were recognised for their participation and awarded prizes in the form of biosecurity equipment (such as buckets, brushes, boots and overcoats) based on their compliance levels. The authors acknowledge that this raises the question of sustainability of the intervention as there is a risk that once incentives are discontinued, farmers will not continue implementing biosecurity on their farms. On the other hand, it is possible that farmers may have improved their compliance due to social pressure for recognition by their peers and local authorities; prioritised improvements from early in the pilot being completed, or genuine buy-in and understanding of potential benefits. In the future, alternative considerations for incentives may include social incentives or incentivising with prizes that are unrelated to biosecurity to avoid bias and promote sustainability or exploring partnerships with the private sector to sponsor such prizes in exchange of publicity.

### 4.4 Strengths and limitations

This study had several strengths and limitations. In particular, the representativeness of the sample of farms is a limitation since non-probability-based sampling was used, due to the list of farms initially provided from the last agricultural census being inaccurate and outdated. When the existing list of pig farmers was validated, many were not present anymore, which is likely related to the fast-changing nature of pig production systems in the selected area. The purposive sampling method may also contribute towards selection bias and impact findings and the sampled farmers should not be considered representative of all the pig farmers in the district. However, to minimise the risk of LFOs proposing farmers that would be more willing to participate, they were blinded from the pilot intervention details before being requested to share sampling frames of pig farms in their respective wards.

More generally, cross-sectional studies are conducted to evaluate the level of biosecurity while evaluation of measures to improve biosecurity are less frequently found in the literature. One exception to this is the farmer field school initiatives (30) although their application in Tanzania is not well documented. Moreover, given the challenges and complexity of performing studies targeting small- and medium-scale farms (such as poor quality and availability of farm-level data and remote study locations) means that the existing evidence-base is limited and renders this study a valuable contribution in the authors’ view. While some of the data collected at the farm-level was from records, good record-keeping was a new concept introduced to the majority of the farmers during this study, and as such, the data collected may suffer from recall bias or obsequiousness bias so findings should be interpreted with some caution. Considering that there are multiple components to the pilot possibly contributing towards increasing adoption of biosecurity measures (sensitisation, provision of incentives, training etc.), without adjusting for confounding, we cannot make inferences that the improvements in on-farm biosecurity observed were due to the checklist only. Observational studies suffer from difficulties in establishing causality, lack appropriate control groups and prospective follow-up periods. When considering conducting an interventional trial, factors such as withholding biosecurity-related extension services were considered ethically unjustifiable in this setting.

## 5 Conclusion

This study demonstrates the potential of a bottom-up approach and several innovative methods, including participatory approaches, blended learning concepts, local-level public-private partnerships and behavioural science to successfully improve biosecurity at farm level. Additionally, these methods can foster trust and strengthen partnerships between farmers and public and private LFOs, which is a crucial prerequisite for achieving meaningful impact on the ground. While the study suggests that improving biosecurity may contribute to reduced mortality, antimicrobial use and improved productivity on pig farms, further research is needed to investigate its long-term impacts, scalability, and sustainability in diverse farming contexts.

As a pilot study, our primary aim was however to test the practical feasibility of these methods, and the insights gained are valuable for other researchers in this field.

The government of Tanzania is currently in contact with FAO to prepare the scaling to neighbouring districts within Rukwa region. Beyond this current effort, we hope that this study inspires others to adopt more participatory approaches, which are applied to various settings and production systems. Strengthening record keeping and data collection is crucial to generate adequate evidence of the benefits resulting from improving biosecurity and can be used to provide motivation to farmers to change their behaviour and promote investments. The findings of this pilot intervention will also be shared to local and national stakeholders to present the case to formally adopt and progressively scale up implementation to other value chain nodes, livestock systems or geographic areas of Tanzania.

By prioritising locally tailored, feasible practices, farmers were empowered to adopt sustainable measures that may improve productivity and reduced morbidity and mortality. The findings highlight the potential of using participatory and bottom-up approaches, combined with sensitization and capacity-building, to address the unique challenges of biosecurity in low-resource settings. Despite limitations such as sample size and resource constraints, this pilot intervention underscores the importance of integrating farmer-led initiatives with local government support. It lays the groundwork for scaling the intervention across other value chains and geographic locations as per the FAO-PMP-TAB framework.

## Data Availability

The raw data supporting the conclusions of this article will be made available by the authors without undue reservation.
