# Supplementary material for: Using a co-created checklist to improve on-farm biosecurity: an observational pilot intervention with pig farmers and livestock field officers in Sumbawanga, Tanzania
Source: Front Vet Sci. 2025 Jul 15;12:1567072. doi: 10.3389/fvets.2025.1567072 (PMC12303800; doi:10.3389/fvets.2025.1567072)
Supplement: Supplementary file 1 [file Data_Sheet_1.docx]

**Supplementary materials**

**Figure 1.** Kobo Collect questionnaire used during farm audits.

Biosecurity checklist - pig farms in Sumbawanga

This checklist provides a set of good practices on biosecurity specifically developed for small and medium-scale pig farmers in Tanzania. These practices apply to ROUTINE biosecurity only, not outbreak situations, which require more rigorous measures led by the local government response. Each practice on the checklist includes examples of compliance to make sure that

practices are easily understood and monitored by extension officers and farmers. The checklist has been developed with local stakeholders. It is not expected that farmers will be compliant to all the practices included on the checklist immediately.

Instead, the checklist provides an approach to progressively improve adoption of good practices step-by-step. The checklist is part of a wider effort by the FAO to pilot the Progressive Management Pathway for Terrestrial Animal Biosecurity (PMP-TAB) in Tanzania. If you have questions or comments about this checklist, please contact Kuboja Lucas (kuboja.mjuberi@fao.org).

# INSPECTION DETAILS (TAARIFA ZA UKAGUZI)

i. Inspector Name ( Jina la mkaguzi)

iii. Date (Tarehe)

yyyy-mm-dd

1. Farm Owner Name ( Jina la mmiliki wa banda)
2. Does farmer have at least 12 months experience in pig farming? Unauzoefu wa ufugaji wa zaidi ya miezi kumi na mbili?


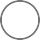

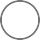
 Yes (Ndio) No (Hapana)

1. Questionnaire respondent (Anaejibu maswali ni)


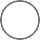
 Farm owner (mmiliki wa shamba)


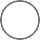
 Farm manager (manager wa shamba)


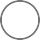
 Other

1. Gender of respondent ( Jinsia)


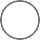

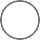
 Male (Mwanaume)
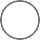
 Women (Mwanamke)

Prefer not to answer

1. ii) Education level of respondent


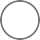
 Primary education
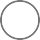
 Secondary education
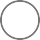
 Diploma


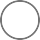
 Bachelors
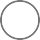
 Postgraduate

1. Farm Location (Eneo banda lilipo)

latitude (x.y °)


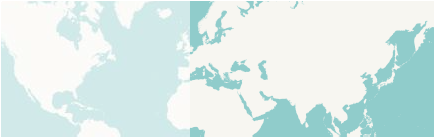

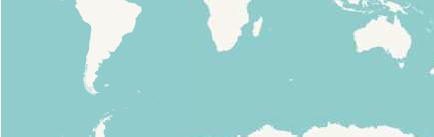


longitude (x.y °)

altitude (m)

accuracy (m)

vii b) Ward name (Kata)

1. Farmer is willing to participate and has signed the community contract (Mfugaji yupo tayari kujiunga na mpango wa majaribio na kuahidi kuboresha mambo ya kwenye orodha hii na ameweka sahihi kwenye mkataba?))


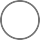
 Yes (Ndio)
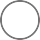
 No (Hapana)

» Farm Production deatails

1. Number of Pigs on farm (Idadi ya Nguruwe)


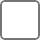
 Sows (Nguruwe Jike)
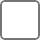
 Boars (Nguruwe dume)


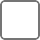
 Finishers (Nguruwe waliokuwa >6months)
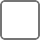
 Growers ( Nguruwe wanaokuwa)


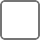
 Piglets (Nguruwe wadogo)

- 1. No. of Sows
  2. No. of Boars
  3. No. of Finishers
  4. No. of Growers
  5. No. of Piglets
  6. Total number of pig on farm ( Jumla ya Nguruwe ni) NaN

1. Type of feed used on farm (choose all that apply) (Aina ya chakula kinachotumika)chagua zote zinazotumika


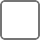
 Commercial feed (pre-formulated and commercially produced feed) (Chakula cha kiwandani)


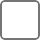
 Homemade feed (own mixing of locally available ingredients) (Chakula kilicho tengenezwa nyumbani)
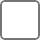
 Swill/kitchen remains (Mabaki ya chakula)


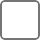
 Other (Nyingine)

1. Use of age-specific feed formulations (Tumia chakula kwa umri)


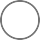
 Yes (Ndio)
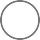
 No (Hapana)

1. Outbreak of ASF in the last month? (Mlipuko ndani ya wiki nne iliyopita?)


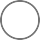
 Yes (Ndio)
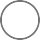
 No (Hapana)

- 1. Month/year of last outbreak (Mwezi/Mwaka wa mlipuko wa mwisho)

yyyy-mm-dd

1. Average number of piglets per litter (on average during past month). IF NO PIGLETS BORN, PLEASE PROVIDE THE AVERAGE (Makadirio ya Idadi ya watoto waliozaliwa kwa uzao mmoja)
2. How many of those piglets usually reach weaning age in the farmer's experience? (Wangapi katika hao nguruwe hufika umri wa kujitegemea kwa uzoefu wako kama mfugaji?)
3. b) How many stillbirths (piglets born dead) in the last month?
4. Number of pigs sold (on average during past month)? (Idadi ya nguruwe waliouzwa ndani ya mwezi uliopita)
5. Number of adult pig deaths (on average during past month)? (Idadi ya vifo kwa wastani ndani ya mwezi uliopita)
6. a) How many ADULT pigs were sick during the last month? ( Je ni nguruwe wangapi waliokomaa walikuwa wagonjwa katika mwezi uliopita?)
7. b) How many PIGLETS were sick during the last month? (Ni nguruwe wangapi wachanga walikuwa wagonjwa katika mwezi uliopita?)

xvii. a) What was the most common cause of sickness? (e.g., diarrhoea, lameness, skin issues) (ni magonjwa gani huwa yanasababisha nguruwe kuumwa? eg kuharisha , kuchechemea, matatizo ya ngozi))

1. Any on farm slaughter of pigs in the last month? (Kuna nguruwe waliochinjwa hapa shambani kwa mwezi uliopita)


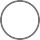
 Yes (Ndio)
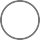
 No (Hapana)

1. At what age (in months) would the farmer usually SELL pigs for slaughter? (Lini mkulima anauza kwa kuchinja?)
2. Estimated final live weight of pigs sold (in kg) in the last month. IF NO PIGS SOLD, PLEASE PROVIDE THE AVERAGE (Makadirio ya uzito wa nguruwe hai kabla ya kuuza ndani ya wiki nne iliyopita (kwa kg))
3. How many times did you use antimicrobials (antibiotics, antiparasitic) in the last month? (Mara ngapi umetumia madawa ya mifugo kwa mwezi ulio pita? (antibayotiki, antiparasiti))
4. How many animals were given antimicrobials in the last month? (Kwa wastani nguruwe wangapi walipewa dawa?)
5. How much (TZS) was spent on antimicrobials in the last month? (Kiasi gani kimetumika kulipia madawa)
6. How many MINUTES were spent working on the farm implementing biosecurity measures? (per day) (Unatumia muda gani (dakika) kufanya matengenezo kwenye shamba lako? (kila siku))

# ON FARM ARRANGEMENTS (MPANGILIO SHAMBANI)

- 1. No visitors allowed without permission (wageni hawaruhusiwi bila ruhusa)

*Fensi inayosunguka shamba; geti; ishara inayoonesha ni eneo lililozuiliwa; wote wanaoingia shambani wanarekodiwa*


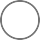
 Yes (Ndio)
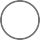
 No (Hapana)

- 1. Keep pigs confined at all times (Nguruwe wafungiwe muda wote)

*Nguruwe haipaswi kuzurura bure; Haipaswi kuwa na mwingiliano kati ya nguruwe na wanyama wengine (k.m., kuku, mbwa, kunguru)*


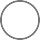
 Yes (Ndio)
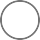
 No (Hapana)

- 1. Changing area before entering pig pen (Eneo la Kubadilishia nguo kabla ya kuingia kwenye zizi la nguruwe)

*Sehemu ya kubadilisha yenye koti na buti (kwa matumizi ndani ya banda la nguruwe PEKEE) ni safi, kavu na kufungwa*


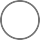
 Yes (Ndio)
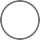
 No (Hapana)

- 1. Change overcoat and boots before entering pens (Badilisha koti na buti kabla ya kuingia kwenye Shamba)

*Buti na nguo maalum za shamba (overcoat) zinazotolewa kwa matumizi ya mazizi ya nguruwe; kubadilisha nguo na viatu baada ya kukutana na wanyama*


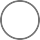
 Yes (Ndio)
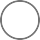
 No (Hapana)

- 1. Segregate pigs by age groups (Tofautisha nguruwe kwa umri )

*Nguruwe wa umri sawa na hatua ya uzalishaji huwekwa / huhifadhiwa pamoja; kati ya vikundi, kalamu safi (kwa kutumia sabuni au sabuni)*

*- ruhusu kukauka - acha tupu kwa siku 14 kabla ya kuanzisha nguruwe wapya*


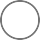
 Yes (Ndio)
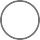
 No (Hapana)

- 1. Good housing structure (Muundo mzuri wa banda la nguruwe)

*Kuta zenye urefu wa kutosha kuzuia nguruwe kuingia kutoka kwenye zizi la karibu; sakafu inayoweza kusafishwa; paa iliyofungwa; kuzuia uwezekano wa ndege (kunguru, kuku) na wanyama wa porini kuingia bandani*


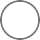
 Yes (Ndio)
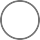
 No (Hapana)

- 1. Good housing conditions (Hali nzuri ya banda la Nguruwe)

*Banda lilotunzwa vizuri: hakuna kuta zilizoharibiwa, sakafu au milango; zizi na sakafu ni safi; uingizaji hewa mzuri ndani ya kalamu*


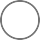
 Yes (Ndio)
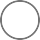
 No (Hapana)

- 1. Access to clean water source (Upatikanaji wa chanzo cha maji safi)

*Maji yanaweza kunywewa, na kutoka kwa vyanzo vya kuaminika (k.m., kutoka kwa usambazaji wa maji wa ndani/kampuni ya umma)*


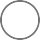

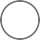
 Yes (Ndio) No (Hapana)

- 1. Animals are handled with care (Wanyama wanahudumiwa kwa uangalifu)

*Nguruwe huonekana katika hali nzuri na safi; mazizi hayajasongamana; epuka kuwasukuma; wafugaji au wafanyikazi wa shamba wanapaswa kuwajibika kuangalia afya ya nguruwe kila siku; angalia watoto wachanga (piglets) kwanza na kisha watu wazima - haswa ikiwa ni mgonjwa au mwenye mashaka au ni mgonjwa*


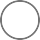
 Yes (Ndio)
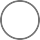
 No (Hapana)

- 1. Clean farm area (Eneo safi kuzunguka banda)

*Hakuna sehemu za taka zinazovutia wanyama wadudu; hakuna maji yaliyotuama au taka zinazovutia nzi*


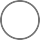
 Yes (Ndio)
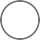
 No (Hapana)

# CLEANING AND DISINFECTION (Kusafisha na kuua viini vya maradhi)

- 1. Washing hands (Kunawa mikono)

*Uwepo wa bomba inayofanya kazi, brashi ya kusugua na sabuni; wafanyakazi na wageni wote wanaosha mikono kwa sabuni kabla ya kuingia kwenye maeneo ya shamba la nguruwe*


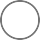
 Yes (Ndio)
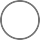
 No (Hapana)

- 1. Cleaning and disinfection (Kusafisha na kuua viini vya maradhi)

*Waendeshaji wanaweza kuonyesha na/au kueleza hatua za kimsingi za mchakato sahihi wa kusafisha na kuua viini (1: Kuondoa uchafu kwa brashi; 2: Osha kwa maji na sabuni; 3: kausha maji 4: Ruhusu ikauke; 5: Weka ua viini vya maradhi baada ya kukauka; 6; Hewa kavu (ili kuhakikisha muda wa kutosha wa dawa kufanya kazi)*


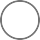
 Yes (Ndio)
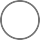
 No (Hapana)

# FEED AND EQUIPMENTS (Chakula na Vifaa)

- 1. No swill feeding (safe feeding) (Hakuna Kulisha mabaki)

*Usilishe nguruwe mabaki ya chakula kutoka jikoni/hoteli/mgahawa ( hasa nyama ya nguruwe) bila kupika kwa angalau dakika 30 kwanza*


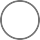
 Yes (Ndio)
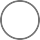
 No (Hapana)

- 1. Feed storage is protected from water, birds, pests or rodents (Hifadhi ya malisho inalindwa dhidi maji, ndege, wadudu au panya)

*Mifuko ya malisho na/au vyombo hufunikwa na kuinuliwa kutoka ardhini; hakuna malisho yaliyomwagika ardhini; hifadhi ya malisho ina lango linaloweza kufungwa*


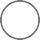
 Yes (Ndio)
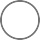
 No (Hapana)

- 1. Use clean farm equipment (Tumia vifaa safi)

*Vifaa vinaonekana ni safi na ni rahisi kusafishwa au kutumia viua vijidudu (hakuna nyufa); epuka kubadilishana vifaa na mashamba mengine na usafishe na kuua vijidudu kabla ya matumizi.*


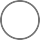

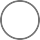
 Yes (Ndio) No (Hapana)

# REPRODUCTION (UZALISHA JI)

- 1. Safe reproduction practices (Uzazi salama)

*Upandishaji wowote wa asili na upandishaji bandia hurekodiwa ikijumuisha tarehe, nguruwe waliopandishwa, nguruwe dume wa kuazima uliotumika na chanzo cha kupata nguruwe dume; kufuga na kutumia nguruwe dume wako mwenyewe - epuka kugawana nguruwe dume isipokuwa kutoka kwa chanzo salama; angalia joto (kwa kutumia kipima joto) la nguruwe dume na nguruwe jike kabla ya kupandana.*

*Usiwapandishe ikiwa halijoto ni>39.5 C; nguruwe jike wapandishwe na nguruwe dume nje ya shamba; nguruwe wanapaswa kutengwa mbali na nguruwe wengine kwa wiki 2 baada ya kupandana*


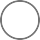
 Yes (Ndio)
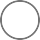
 No (Hapana)

# WASTE DISPOSAL

- 1. Solid animal waste is safely disposed on daily basis(Taka ngumu za wanyama kutupwa kwa usalama kila siku)

*Taka hutolewa mara moja kutoka kwa zizi ili kuzuia uchafuzi; hakuna taka zinazotupwa nje ya makazi ya nguruwe, karibu na shamba au juu ya uzio*


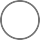

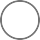
 Yes (Ndio) No (Hapana)

- 1. Good drainage on-farm (liquid waste disposal) (Mifereji bora ya maji shambani (utupaji wa taka za maji) )

*Matumizi ya mifereji bora ya maji ili kuhakikisha mtiririko mzuri wa maji na taka; uwepo wa shimo la uchafu; taka za maji (pamoja na damu ya nguruwe) hazimwagiki kwenye mazingira*


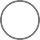
 Yes (Ndio)
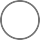
 No (Hapana)

- 1. Safe carcass disposal (Sehemu salama ya kutupa mizoga)

*Fukia chini au choma mizoga; hufanywa sehemu salama yenye uzio au inayozuia kufikika kwa nguruwe wengine na wanyama wawindaji.*


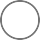
 Yes (Ndio)
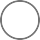
 No (Hapana)

# NEW ANIMALS AND SICK ANIMALS (WANYAMA WAPYA NA WANYAMA WAGONJWA )

- 1. Only purchase disease-free, healthy pigs (Nunua Nguruwe wasio na ugonjwa na wenye afya njema)

*Ikiwa kununuliwa, nguruwe inapaswa kutoka kwa vyanzo visivyo na magonjwa au nguruwe wanaonekana kuonekana kuwa na afya*


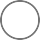
 Yes (Ndio)
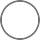
 No (Hapana)

- 1. Isolate new pigs (introductions) and sick pigs (Tenga nguruwe wapya na nguruwe wagonjwa)

*Tenganisha banda mbali la nguruwe wenye afya nzuri na nguruwe wapya. Tenga utangulizi mpya kwa siku 14; tofauti vifaa na nguo za kuhudumia nguruwe wagonjwa*


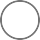
 Yes (Ndio)
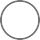
 No (Hapana)

- 1. No movement or sale of sick or infected pigs(Hakuna kuzurura au uuzaji wa nguruwe wagonjwa au walioambukizwa)

*Hakuna kutoa nguruwe wagonjwa, haswa wakati wa kuzuka; nguruwe wagonjwa au walioambukizwa wanapaswa kutengwa au kutengwa bila kupelekwa sokoni au kuuzwa kwa bei ya chini*


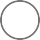

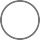
 Yes (Ndio) No (Hapana)

- 1. Report sick or infected pigs to veterinary services(Ripoti nguruwe wagonjwa au walioambukizwa kwa huduma za mifugo )

*Rekodi zinaonndioha kuripoti kulitokea ndani ya masaa 24 baada ya ishara kutambuliwa*


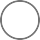
 Yes (Ndio)
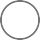
 No (Hapana)

- 1. Continuous training about good animal husbandry (Mafunzo ya ufugaji bora)

*Mfugaji ni rahisi kujibu maswali ya kimsingi (k.m. umuhimu wa usalama wa viumbe hai, ni nini dalili kuu ya ugonjwa wa homa ya nguruwe?; wakati wa kutumia antibiotics; ni joto gani la kawaida la nguruwe aliyekomaa; ni ugonjwa gani unaoweza kutambuliwa, nk.)*


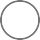
 Yes (Ndio)
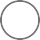
 No (Hapana)

# RECORD KEEPING (UTUNZA JI KUMBUKUMBU)

- 1. Use record keeping system(Tumia mfumo wa kutunza kumbukumbu )

*Rekodi zinapatikana na ni za kisasa; tumia templeti au kitabu cha kumbukumbu ya majaribio ya mradi wa PMP-TAB*


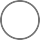
 Yes (Ndio)
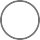
 No (Hapana)

# USE OF VETERINARY DRUGS ( MATUMIZI YA MADAWA YA MIFUGO)

- 1. Prudent use of veterinary drugs (Matumizi bora ya madawa ya mifugo)

*Madawa yahifadhiwe eneo moja; madawa yote ya kutibu na chanjo lazima yatolewe kwa ushauri wa Mtaalam wa mifugo; kutunza kumbukumbu za matumizi ya ya madawa*


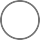
 Yes (Ndio)
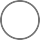
 No (Hapana)

Your total compliance score is 0.

Your total compliance percentage is 0

1. Any additional comments/observations (Maoni/Ufafanuzi wa ziada)

Add photo of housing conditions (weka picha - banda)

Click here to upload file. (< 10MB)

1. Which out of the 26 practices is the respondent willing to improve before the next visit? Please include at least 1 or a maximum of 3 practices (ni jambo gani ungependa kuboresha kati ya hayo 26 hapo juu?)
   1.
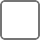
No visitors allowed without permission (Hakuna wageni wanaoruhusiwa bila ruhusa)
   2.
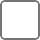
Keep pigs confined at all times (Nguruwe wafungiwe mda wote)
   3.
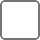
Changing area before entering pig pen (Eneo la Kubadilishia nguo kabla ya kuingia kwenye zizi la nguruwe)
   4.
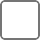
Change overcoat and boots before entering pens
   5.
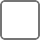
Segregate pigs by age groups (Tofautisha nguruwe kwa vikundi vya umri)
   6.
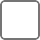
Good housing structure (Muundo mzuri wa banda la nguruwe)
   7.
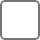
Good housing conditions (Hali nzuri ya banda la nguruwe)
   8.
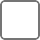
Access to clean water source (Upatikanaji wa chanzo cha maji safi)
   9.
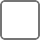
Animals are handled with care (Wanyama wanahudumiwa kwa uangalifu)
   10.
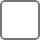
Clean farm area (Eneo safi kuzunguka banda)
   11.
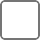
Washing hands (Kuosha mikono)
   12.
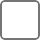
Cleaning and disinfection (Kusafisha na kuua viini vya maradhi)
   13.
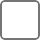
No swill feeding (safe feeding) (Hakuna kilisha mabaki)
   14.
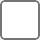
Feed storage is protected from water, birds, pests or rodents (Hifadhi ya malisho inalindwa dhidi maji, ndege, wadudu au panya)
   15.
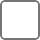
Use clean farm equipment (Tumia vifaa safi)
   16.
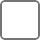
Safe reproduction practices (Uzazi salama)
   17. Solid animal waste is safely and promptly disposed (Taka ngumu za wanyama hutupwa kwa usalama na mara moja)
   18. Good drainage on-farm (liquid waste disposal) (Mifereji bora ya maji shambani (utupaji wa taka za maji))
   19. Safe carcass disposal (Sehemu salama ya kutupa mizoga)
   20. Only purchase disease-free, healthy pigs (Nunua nguruwe wasio na ugonjwa na wenye afya njema)
   21. Isolate new pigs and sick pigs (Tenga nguruwe wapya na nguruwe wagonjwa)
   22. No movement or sale of sick or infected pigs (Hakuna kutoa au uuzaji wa nguruwe wagonjwa au walioambukizwa)
   23. Report sick or infected pigs to veterinary services (Ripoti nguruwe wagonjwa au walioambukizwa kwa huduma za mifugo)
   24. Training on good animal husbandry (Mafunzo ya ufugaji bora)
   25. Use record keeping system (Tumia mfumo wa kutunza kumbukumbu)
   26. Prudent use of veterinary drugs (Matumizi bora ya madawa ya mifugo)

What is the main objective of the farmer to invest in biosecurity?

*This is an open-ended question.*

Please select the FIRST biosecurity practice you are focusing on based on the farmer you are interviewing (check the sheet provided).

1. No visitors allowed without permission (Hakuna wageni wanaoruhusiwa bila ruhusa)
2. Keep pigs confined at all times (Nguruwe wafungiwe mda wote)
3. Changing area before entering pig pen (Eneo la Kubadilishia nguo kabla ya kuingia kwenye zizi la nguruwe)x
4. Change overcoat and boots before entering pens
5. Segregate pigs by age groups (Tofautisha nguruwe kwa vikundi vya umri)
6. Good housing structure (Muundo mzuri wa banda la nguruwe)
7. Good housing conditions (Hali nzuri ya banda la nguruwe)
8. Access to clean water source (Upatikanaji wa chanzo cha maji safi)
9. Animals are handled with care (Wanyama wanahudumiwa kwa uangalifu)
10. Clean farm area (Eneo safi kuzunguka banda)
11. Washing hands (Kuosha mikono)
12. Cleaning and disinfection (Kusafisha na kuua viini vya maradhi)
13. No swill feeding (safe feeding) (Hakuna kilisha mabaki)
14. Feed storage is protected from water, birds, pests or rodents (Hifadhi ya malisho inalindwa dhidi maji, ndege, wadudu au panya)
15. Use clean farm equipment (Tumia vifaa safi)
16. Safe reproduction practices (Uzazi salama)
17. Solid animal waste is safely and promptly disposed (Taka ngumu za wanyama hutupwa kwa usalama na mara moja)
18. Good drainage on-farm (liquid waste disposal) (Mifereji bora ya maji shambani (utupaji wa taka za maji))
19. Safe carcass disposal (Sehemu salama ya kutupa mizoga)
20. Only purchase disease-free, healthy pigs (Nunua nguruwe wasio na ugonjwa na wenye afya njema)
21. Isolate new pigs and sick pigs (Tenga nguruwe wapya na nguruwe wagonjwa)
22. No movement or sale of sick or infected pigs (Hakuna kutoa au uuzaji wa nguruwe wagonjwa au walioambukizwa)
23. Report sick or infected pigs to veterinary services (Ripoti nguruwe wagonjwa au walioambukizwa kwa huduma za mifugo)
24. Training on good animal husbandry (Mafunzo ya ufugaji bora)
25. Use record keeping system (Tumia mfumo wa kutunza kumbukumbu)
26. Prudent use of veterinary drugs (Matumizi bora ya madawa ya mifugo)

Let's talk about [selected practice]. Why is this necessary?

*Can the farmer explain why the biosecurity practice is necessary on their farm? (I.e., can they explain the benefits?)*

No knowledge Little knowledge Some knowledge

Extensive knowledge

How confident are you in your ability to implement [selected practice]?

Not confident

Somewhat confident Very confident

Do you ever forget [selected practice]?

Never Rarely

Sometimes Often

Always

Do you have all the resources available to implement [selected practice]?

No resources

Limited resources

All resources are available

What specific resources are you lacking?

*This is an open-ended question.*

What do people whose opinions you value think about implementing [selected practice]?

They strongly encourage it They somewhat support it They are neutral or indifferent They somewhat discourage it They strongly discourage it

How common is [selected practice] amongst the pig farmers in your community?

Very common Common

Somewhat common Uncommon

Very uncommon

How motivated are you to continue or begin [selected practice]?

Not at all motivated Somewhat motivated Very motivated

Do you feel rewarded when you have implemented [selected practice]?

Yes No

Please explain how you feel/don't feel rewarded.

*This is an open-ended question.*

Are you concerned when you skip or don't implement [selected practice]?

Not at all concerned Somewhat concerned Very concerned

How do you feel when you follow/implement [selected practice]?

Very positive Positive

Neutral

Slightly negative Very negative

How optimistic are you that [selected practice] will benefit your farming business?

Not at all optimistic Somewhat optimistic Very optimistic

Is [selected practice] something you do out of habit (without even thinking about it)?

Never Rarely

Sometimes Often

Always

How important is [selected practice] to your role as a livestock farmer?

Not at all important Somewhat important Very important

What are some challenges to implementing [selected practice]?

*This is an open-ended question. Please note if the farmer replies that there are no challenges.*

**Table 1**. Pig farmers total biosecurity compliance score (out of 26 practises) at baseline and the endline of the intervention stratified by demographic and farm characteristics (n=28)*.

| **Variable** | **Category** | **Baseline (before)** | | | | | **After** | | | | |
| --- | --- | --- | --- | --- | --- | --- | --- | --- | --- | --- | --- |
|  |  | Mean | Median | SD | Min | Max | Mean | Median | SD | Min | Max |
| **Gender** | Male | 6.0 | 5.5 | 4.5 | 1.0 | 17.0 | 18.2 | 20.0 | 6.4 | 5.0 | 26.0 |
|  | Female | 6.8 | 7.0 | 5.0 | 1.0 | 17.0 | 21.1 | 21.5 | 3.6 | 14.0 | 25.0 |
| **Education level** | Primary education | 3.5 | 2.0 | 2.9 | 1.0 | 10.0 | 15.2 | 17.0 | 6.1 | 5.0 | 23.0 |
|  | Secondary education | 7.0 | 6.0 | 6.4 | 1.0 | 17.0 | 17.4 | 20.0 | 8.0 | 7.0 | 25.0 |
|  | Diploma | 6.9 | 6.0 | 4.9 | 2.0 | 17.0 | 21.3 | 22.0 | 3.7 | 14.0 | 25.0 |
|  | Bachelors | 9.0 | 9.0 | 0.0 | 9.0 | 9.0 | 24.5 | 24.5 | 2.1 | 23.0 | 26.0 |
|  | Postgraduate | 12.0 | 12.0 | N/A | 12.0 | 12.0 | 25.0 | 25.0 | N/A | 25.0 | 25.0 |
| **Herd size** | 0-5 | 4.3 | 5.0 | 2.3 | 1.0 | 7.0 | 20.2 | 20.0 | 2.0 | 17.0 | 22.0 |
|  | 6-10 | 4.2 | 2.0 | 3.6 | 1.0 | 9.0 | 14.4 | 14.0 | 7.1 | 7.0 | 23.0 |
|  | 11-20 | 5.1 | 4.0 | 3.8 | 1.0 | 12.0 | 15.6 | 14.0 | 7.0 | 5.0 | 25.0 |
|  | 21-30 | 8.3 | 5.0 | 7.6 | 3.0 | 17.0 | 22.3 | 23.0 | 2.1 | 20.0 | 24.0 |
|  | 31-40 | 8.3 | 9.0 | 2.1 | 6.0 | 10.0 | 23.5 | 23.5 | 2.1 | 22.0 | 25.0 |
|  | 41-50 | - | - | - | - | - | - | - | - | - | - |
|  | >50 | 13.0 | 13.0 | 5.7 | 9.0 | 17.0 | 25.5 | 25.5 | 0.7 | 25.0 | 26.0 |
| **ASF outbreak**** | Yes | 8.0 | 7.0 | 6.0 | 1.0 | 17.0 | 20.1 | 23.0 | 5.0 | 14.0 | 25.0 |
|  | No | 5.6 | 5.0 | 4.0 | 1.0 | 17.0 | 17.7 | 20.0 | 6.6 | 5.0 | 26.0 |
| **Ward** | Chanji | 6.0 | 6.0 | 3.4 | 2.0 | 10.0 | 20.7 | 22.0 | 3.2 | 17.0 | 23.0 |
|  | Kasense | 4.5 | 4.0 | 4.1 | 1.0 | 9.0 | 17.8 | 20.0 | 8.9 | 5.0 | 26.0 |
|  | Kizwite | 12.0 | 10.0 | 4.4 | 9.0 | 17.0 | 24.0 | 24.0 | 2.0 | 23.0 | 25.0 |
|  | Lwiche | 6.0 | 6.0 | 0.0 | 6.0 | 6.0 | 20.0 | 20.0 | 0.0 | 20.0 | 20.0 |
|  | Majengo | 2.7 | 3.0 | 1.5 | 1.0 | 4.0 | 15.3 | 13.0 | 5.9 | 11.0 | 22.0 |
|  | Mollo | 5.7 | 4.0 | 5.7 | 1.0 | 12.0 | 15.7 | 14.0 | 8.6 | 8.0 | 25.0 |
|  | Momoka | 7.0 | 7.0 | N/A | 7.0 | 7.0 | 10.0 | 10.0 | N/A | 10.0 | 10.0 |
|  | Ntendo | 2.8 | 2.0 | 2.4 | 1.0 | 7.0 | 14.2 | 14.0 | 7.3 | 7.0 | 25.0 |
|  | Pito | 8.8 | 8.0 | 4.9 | 5.0 | 17.0 | 22.8 | 22.5 | 1.7 | 21.0 | 25.0 |

* Due to incompleteness of data and inadequate counts, no statistical testing was carried out.
** ASF outbreak reported in the 6 months prior to the pilot start date.
